# Likelihood of changes in forest species suitability, distribution, and diversity under future climate: The case of Southern Europe

**DOI:** 10.1002/ece3.3427

**Published:** 2017-10-07

**Authors:** Sergio Noce, Alessio Collalti, Monia Santini

**Affiliations:** ^1^ Foundation Euro‐Mediterranean Center on Climate Change (CMCC) – Impacts on Agriculture, Forests and Ecosystem Services (IAFES) Division Viterbo Italy; ^2^ Department for Innovation in Biological, Agro‐food and Forest systems (DIBAF) University of Tuscia Viterbo Italy; ^3^ CNR‐ISAFOM – National Research Council of Italy Institute for Agriculture and Forestry Systems in the Mediterranean Rende Italy; ^4^ Far East Federal University (FEFU) Vladivostok Russia

**Keywords:** bioclimatic predictors, BIOMOD, climate change, forest diversity, forests, geographic information systems, global circulation models, likelihood

## Abstract

Forest conservation strategies and plans can be unsuccessful if the new habitat conditions determined by climate change are not considered. Our work aims at investigating the likelihood of future suitability, distribution and diversity for some common European forest species under the projected changes in climate, focusing on Southern Europe. We combine an Ensemble Platform for Species Distribution Models (SDMs) to five Global Circulation Models (GCMs) driven by two Representative Concentration Pathways (RCPs), to produce maps of future climate‐driven habitat suitability for ten categories of forest species and two time horizons. For each forest category and time horizon, ten maps of future distribution (5 GCMs by 2 RCPs) are thus combined in a single suitability map supplied with information about the “likelihood” adopting the IPCC terminology based on consensus among projections. Then, the statistical significance of spatially aggregated changes in forest composition at local and regional level is analyzed. Finally, we discuss the importance, among SDMs, that environmental predictors seem to have in influencing forest distribution. Future impacts of climate change appear to be diversified across forest categories. A strong change in forest regional distribution and local diversity is projected to take place, as some forest categories will find more suitable conditions in previously unsuitable locations, while for other categories the same new conditions will become less suited. A decrease in species diversity is projected in most of the area, with Alpine region showing the potentiality to become a refuge for species migration.

## 1 INTRODUCTION

Global change poses a great challenge to the actors (policy makers and stakeholders) who deal with ecosystem protection and conservation strategies (Brooks et al., 2006; Hannah & Midgley, 2002; Hannah et al., 2002). Traditional conservation approaches, which are mainly based on existing and static protected areas, may be totally ineffective if they neglect the impacts of the changing environmental, especially climatic, conditions (Le Saout et al., 2013; Leroux & Rayfield, 2014; Ochoa‐Ochoa, Flores‐Villela, & Bezaury‐Creel, 2016; Rayfield, James, Fall, & Fortin, 2008; Socha, Coops, & Ochal, 2016). Future conservation strategies and plans play a key role in preserving ecosystems against both natural hazards and human threats, in particular due to environmental resources’ exploitation and land‐use changes. Therefore, in delineating these strategies and plans, the concepts of biogeography and expected shifts in species habitats, as well as the modification in species regional distribution and local diversity under climate projections, become critical (Araújo, Alagador, Cabeza, Nogués‐Bravo, & Thuiller, 2011; Araujo, Cabeza, Thuiller, Hannah, & Williams, 2004; Bakkenes, Alkemade, Ihle, Leemans, & Latour, 2002; Guisan et al., 2013; Kelly & Goulden, 2008; Serra‐Diaz, Scheller, Syphard, & Franklin, 2015). For instance, while a recent study on forest presence hot spots demonstrated the local value of protected areas in southern Europe (Noce, Collalti, Valentini, & Santini, 2016), these areas do not guarantee the same benefits under future environmental scenarios (Araújo et al., 2011). It is therefore crucial to recognize the role of biogeography and of the factors affecting ecosystems’ composition and biodiversity at scale of species ranges over time (Franklin, 2016).

Forest ecosystems are strategic for biodiversity conservation; on a centennial scale, they have evolved their resilience and adaptation capability to disturbances (e.g., droughts, fires, windstorms, pests, diseases, and invasive species), including migration as an option (Aitken, Yeaman, Holliday, Wang, & Curtis‐McLane, 2008). Under global change and, thus, with an altered intrinsic vulnerability due to modified average environmental conditions, forest communities have to face an additional challenge: coping with a quickly increasing variability of extreme events and disturbances (Seidl, Spies, Peterson, Stephens, & Hicke, 2016) as well as novel perturbations (e.g., new diseases; Pautasso, Schlegel, & Holdenrieder, 2015). Such complex transformations are occurring too fast to be accompanied by both evolutionary adaptation (Dale et al., 2001; Lindner et al., 2010; Trumbore, Brando, & Hartmann, 2015) and migration processes, although the latter seems the best option for species (Corlett & Westcott, 2013). It is widely demonstrated that the geographic distribution of forest species is strictly correlated with medium‐ to long‐term climate conditions (Araújo & Pearson, 2005; Park Williams et al., 2012; Schimper & Fisher, 1903), topographic factors (Bellingham & Tanner, 2000) and, especially in the Mediterranean region, human activities (Barbero, Bonin, Loisel, & Quézel, 1990). These elements, when combined, realize the concept of niche (Guisan & Zimmermann, 2000).

Correlative Species Distribution Models (SDMs) that are also known as bioclimatic envelope models, correlative ecological niche models, or habitat suitability models, explore the relationships and the equilibrium between the geographical distribution of species and a set of environmental variables (Austin, 2002; Guisan & Zimmermann, 2000; Naimi & Araújo, 2016; Peterson et al., 2011).

SDMs, in conjunction with Geographic Information Systems (GIS) tools, are promising research tools to map and predict the potential spread of endemic or invasive species in the past and the future, respectively (Franklin, 2010). Thanks to these tools, scientists are in fact enabled to inform decision makers and stakeholders on how to formulate or prioritize biodiversity and biogeography conservation plans and to contribute to the maintenance of multiple services provided by forests, including the mitigation of climate change and its impacts (Franklin, 2013; Gama, Crespo, Dolbeth, & Anastácio, 2015).

Nevertheless, SDM approach has some limitations in particular related to unavoidable assumptions and uncertainties (Guisan & Thuiller, 2005; Watling et al., 2015). The most important element concerns the selection of environmental explanatory factors (or predictors) (Lexer & Hönninger, 2004). Often this choice is constrained by the availability of datasets (that also have their own uncertainty as stated in Bedia, Herrera, & Gutierrez, 2013), or by the suspicion of redundancy among predictors that seem correlated (collinear). Unfortunately, any mechanistic and ecological understanding of the “true” predictors still lacks of interpretability (Dormann et al., 2013). Even in case of dealing with collinearity, adopting one or another method presents different uncertainties (Dormann, Purschke, Márquez, Lautenbach, & Schröder, 2008). Moreover, the assumption that the relationships between predictors and species presence/absence assessed for the historical period will maintain in the future could be really uncertain (Gavin et al., 2014; Hijmans & Graham, 2006). Moreover, a modeled species is rarely observed in its full climate space, and usually model performances are tested just in a “restricted” climate space (Hannemann, Willis, & Macias‐Fauria, 2016) and within boundaries smaller than physiological limits of species (Loehle & LeBlanc, 1996), especially in regional studies; this is also due to the limited availability of harmonized species distribution data. Another major limitation is that SDMs do not routinely consider relevant population and dispersal dynamics or intraspecific variation in climatic tolerances (Holt, 2009; Zurell et al., 2016). Summarizing, results are widely dependent on: (i) the reliability and accuracy of species occurrence data; (ii) the significance of the environmental variables selected; (iii) the quality of related data; and (iv) the parameterization or configuration of the applied models (Chakraborty et al., 2016; Nenzén & Araújo, 2011; Thuiller, 2003; Thuiller, Lafourcade, Engler, & Araújo, 2009). Given that all above elements cause a large variability in the predictions (Cheaib et al., 2012; Pearson et al., 2006; Thuiller et al., 2004), the Ensemble Forecasting approach has been developed and widely adopted (Araujo & New, 2007; Heikkinen et al., 2006; Komac, Esteban, Trapero, & Caritg, 2016; Marmion, Parviainen, Luoto, Heikkinen, & Thuiller, 2009). This approach combines individual SDM predictions to provide consensus predictions (Capinha & Anastácio, 2011), enabling more robust evaluations, that is, addressing the uncertainty related to SDMs.

After assessing the accuracy of the Ensemble Forecasting‐SDM approach in reproducing the historical species distributions, results for future projections mainly depend on the updated values of the considered dynamic predictors (i.e., changing at the time scale of simulations). These are usually bioclimatic variables calculated from the outputs of climate model simulations that, in turn, are driven by scenarios on greenhouse gas (GHG) emissions or concentrations. Considering a single projection is, however, not recommended, indeed, as highlighted by Lindner et al. (2014), the scientific community cannot still accurately forecast GHG emissions and the ways in which the climate will evolve.

The Coupled Model Intercomparison Project 5 (CMIP5), which informed the Fifth Assessment Report by the Intergovernmental Panel on Climate Change (IPCC‐AR5), provides a framework of coordinated climate experiments conducted through Global Circulation and Earth System Models. The CMIP5 outputs are anyway characterized by a high level of uncertainty (Friedlingstein et al., 2014; Taylor, Stouffer, & Meehl, 2012) due to the models’ physics, initialization and/or configurations, increased by the consideration of four different representative concentration pathways (RCPs) (van Vuuren et al., 2011). Such variability in climate projections needs to be considered, quantified, and well managed, especially when used in concatenated impacts evaluations, so to explore a broad range of possible developments (Cheaib et al., 2012).

Further studies (Goberville, Beaugrand, Hautekèete, Piquot, & Luczak, 2015; Keenan, Maria Serra, Lloret, Ninyerola, & Sabaté, 2011; Lindner et al., 2008; Wang, Campbell, O'Neill, & Aitken, 2012) concerning climate change and impacts on European forests focused the attention on the large uncertainty and lack of knowledge related to future distribution scenarios. It is thus clear that climate change impact evaluations could be used for planning and decisions only after assessing the uncertainty or, to more directly inform stakeholders and policy makers, after treating the uncertainty by quantifying the likelihood of outcomes based on the agreement among multiple simulations.

In this work, we adopt the Ensemble Forecasting‐SDM approach to predict the possible impacts of climate change in terms of geographic range shifts, over medium and long term, for ten forest categories (groups of species). We focus our attention on southern Europe, and the Mediterranean Basin in particular, as they are among the world's major areas for plant biodiversity and endemism (Medail & Quezel, 1997, 1999; Myers, Mittermeier, Mittermeier, daFonseca, & Kent, 2000; Underwood, Viers, Klausmeyer, Cox, & Shaw, 2009). These regions are dominated by a Warm Temperate climate that ranges from dry and warm to hot summers (Alessandri et al., 2014; Kottek, Grieser, Beck, Rudolf, & Rubel, 2006), and they are projected, with a high degree of consistency among different projections, as hot spots of climate change under IPCC‐AR4 scenarios (Giorgi, 2006). In these areas, raising temperature and decreasing summer precipitations will lead to an increase in summer droughts (Giorgi & Lionello, 2008; Mariotti et al., 2008). In terms of average climate, the robust assessment (i.e., derived by probabilistic ensemble evaluation) in Alessandri et al. (2014) suggests that this climate is expected to experience a northward shift under IPCC‐AR5 intermediate (RCP4.5) emission scenario, leaving space for more arid conditions as also evinced in Santini and di Paola (2015). Previous impact studies like those in Santini, Collalti, and Valentini (2014) also show the consensus in predicting an increase of water scarcity and fire disturbances in this region, with negative consequences for forest ecosystems.

Other studies (Duputié, Zimmermann, & Chuine, 2014; Zimmermann, Jandl et al., 2013), and the JRC “Tree Species Maps—Species Habitat Suitability” dataset (http://forest.jrc.ec.europa.eu/download/data/species-distribution/) in particular, give useful information about the expected forest suitability in the European area. Our work aims to complement these efforts by merging/harmonizing two datasets of forest species presence (Noce et al., 2016), and exploiting the latest projections under CMIP5, guaranteeing robustness in terms of model consensus in predicting future distribution of each forest category. We use the IPCC terminology on likelihood to treat the uncertainty on future outlooks (Mastrandrea et al., 2011). The approach we adopt is what we call a *cascade ensemble system*, which concatenates the ensemble forecasting approach of SDMs to a sub‐ensemble of CMIP5 climate projections.

Further, we investigate, for the whole study area and for its main bio‐geographical subregions, the projected future evolution in terms of forest spatial arrangement (distribution) and local species diversity, which is part of the wider biodiversity concept (Boudouresque, 2011). We then compare and discuss the overall importance, averaged across model, of bioclimatic predictors at species level, corroborated with some ecological and mechanistic considerations based on knowledge of historical habitat distribution. The results presented here can be helpful for further ecological and biodiversity conservation evaluations as well as support of medium‐ to long‐term protection or restoration strategies that account for climate change and its uncertainty range.

## 2 MATERIALS AND METHODS

**Figure 1:**
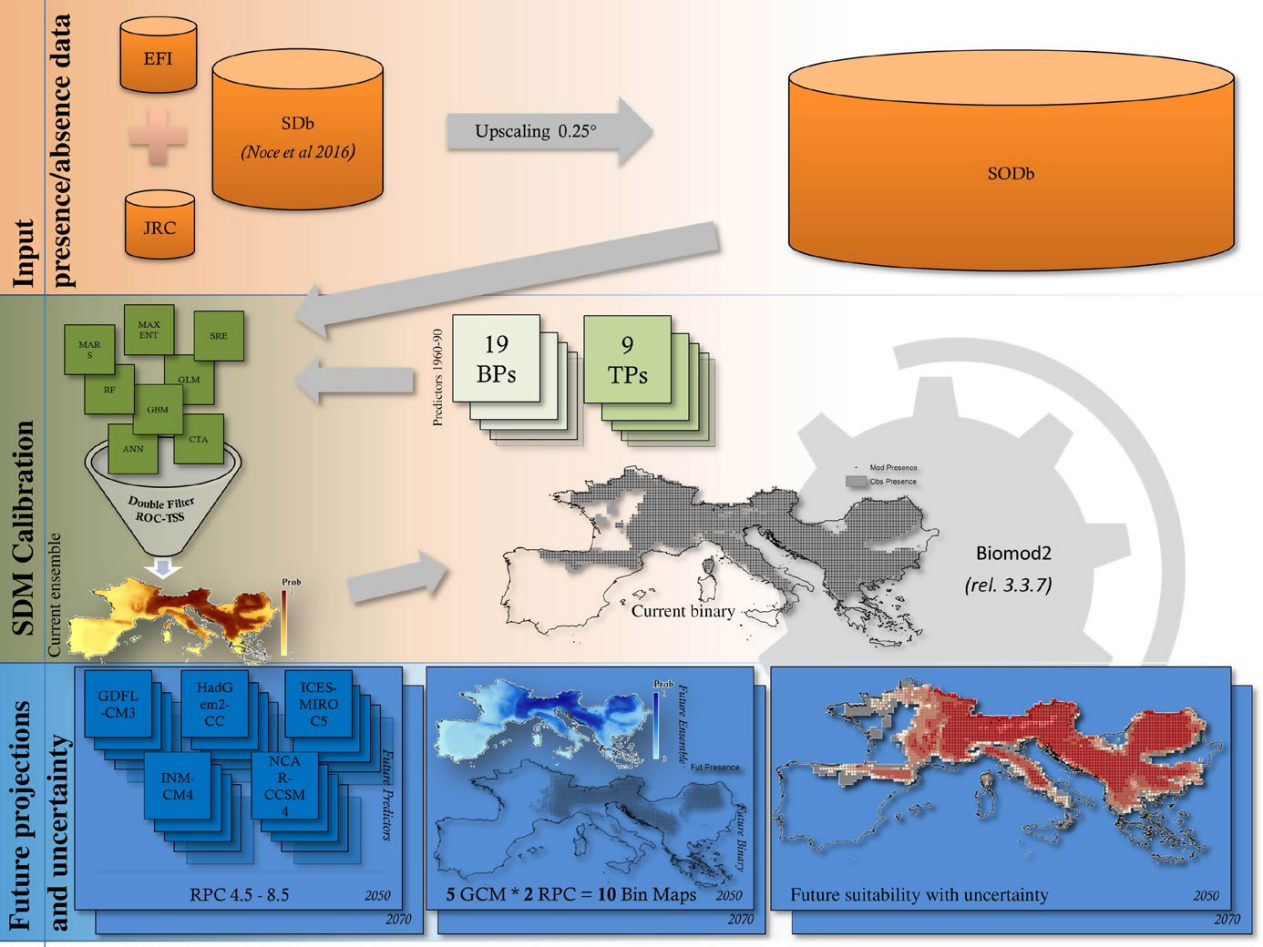
Graphical overview of methods

In the following paragraphs data and methods are described, while Fig. 1 provides a graphical overview of the overall approach.

### 2.1 Species distribution modeling

The potential distribution of the considered tree forest categories under climate change was projected through the “BIOMOD2” package v3.3‐7 (https://cran.r-project.org/web/packages/biomod2/index.html; Thuiller, Georges, Engler, & Breiner, 2016) implemented in R (R Core Team, 2015). The BIOMOD2 platform uses individual species representation and a community‐based approach, enabling ensembles of models to be evaluated (calibration phase) with the historical conditions (represented by environmental predictors) and then re‐applied to project the potential spatial distributions (habitat suitability driven by climate) of species along future time horizons (Naimi & Araújo, 2016).

Ten models are embedded in BIOMOD2 and include the following: two regression methods, which build linear or nonlinear relationships between species occurrence and their environmental predictors (Generalized Linear Models—GLMs and Generalized Additive Models—GAMs); five machine‐learning or complex methods, that extract the environmental space of the species occurrence directly from the training predictors’ data (Artificial Neural Networks—ANN, Boosted Regression Trees or Generalized Boosted model—BRT/GBM, Multivariate Adaptive Regression Splines—MARS, Maximum Entropy—MaxEnt, and Random Forest—RF); two classification methods, which are based on successive partitioning of predictors’ data into homogeneous groups of response (Classification and Regression Trees or Classification Tree Analysis—CART or CAT—and Flexible discriminate Analysis—FDA); and finally a Surface Range Envelope (SRE) method, which defines the environmental conditions of the species occurrence and extrapolates the results to similar areas (Duque‐Lazo, van Gils, Groen, & Navarro‐Cerrillo, 2016). As stated by Ochoa‐Ochoa et al. (2016), the combined use of both simple and complex methods is beneficial as there is not yet a clear better performance of one or the other method, and it depends on the region or the quality of the data.

BIOMOD2 also offers a set of rules and metrics for SDM evaluations, based on the confusion matrix and its elaborations (Miller, 2010) into three metrics: Area Under the Curve of the Receiver Operating Characteristic plot (ROC), Cohen's K and True Skills Statistics (TSS) (Duque‐Lazo et al., 2016). ROC is a threshold‐independent model evaluation indicator (Franklin, 2010), and it is also independent of prevalence (i.e., the frequency of occurrence) of target species. It plots the commission error (1—specificity; false positives) against omission error (sensitivity; true positives). ROC ranges between 0.5–1, where 1 represents a perfect discrimination between presence and absence, and 0.5 represents a random fit. Both Cohen's K and TSS are threshold‐dependent measures of model accuracy. They both range from −1 to +1, with +1 indicating perfect agreement between predictions and observations, and 0 or less indicating an agreement no better than a random classification (Zhang et al., 2015). Cohen's K has been criticized as being strongly influenced by the species prevalence in the data, and the TSS has been introduced to solve this problem (Allouche, Tsoar, & Kadmon, 2006).

Regardless of the evaluation metrics, model should always be evaluated by means of independent data or adopting data splitting procedures. In BIOMOD2, a user‐defined proportion of the original data can be used for training the models, while the remaining proportion is used for model evaluation. The package also allows conducting *n* data splitting runs, providing an *n*‐fold cross‐validation procedure. Only runs that meet criteria in terms of evaluation metrics are included in the ensemble, and weighted according to their performances before to generate the final binary (presence/absence) map.

In addition to model evaluation, the importance of selected and tested predictors can be analyzed. Once the models are trained (i.e., calibrated), a standard prediction is made. Afterwards, one of the variables is randomized, and a new prediction is made. The correlation score between the new prediction and the standard prediction is considered to give an estimation of the variable importance in the model. A good correlation score between the two predictions suggests that the randomized variable has little importance. On the contrary, a low correlation means a significant difference in the predictions, making that variable important for the model. Importance is expressed as 1 minus correlation and converted to percentage for easier interpretation.

### 2.2 Study area and forest occurrence dataset

**Figure 2:**
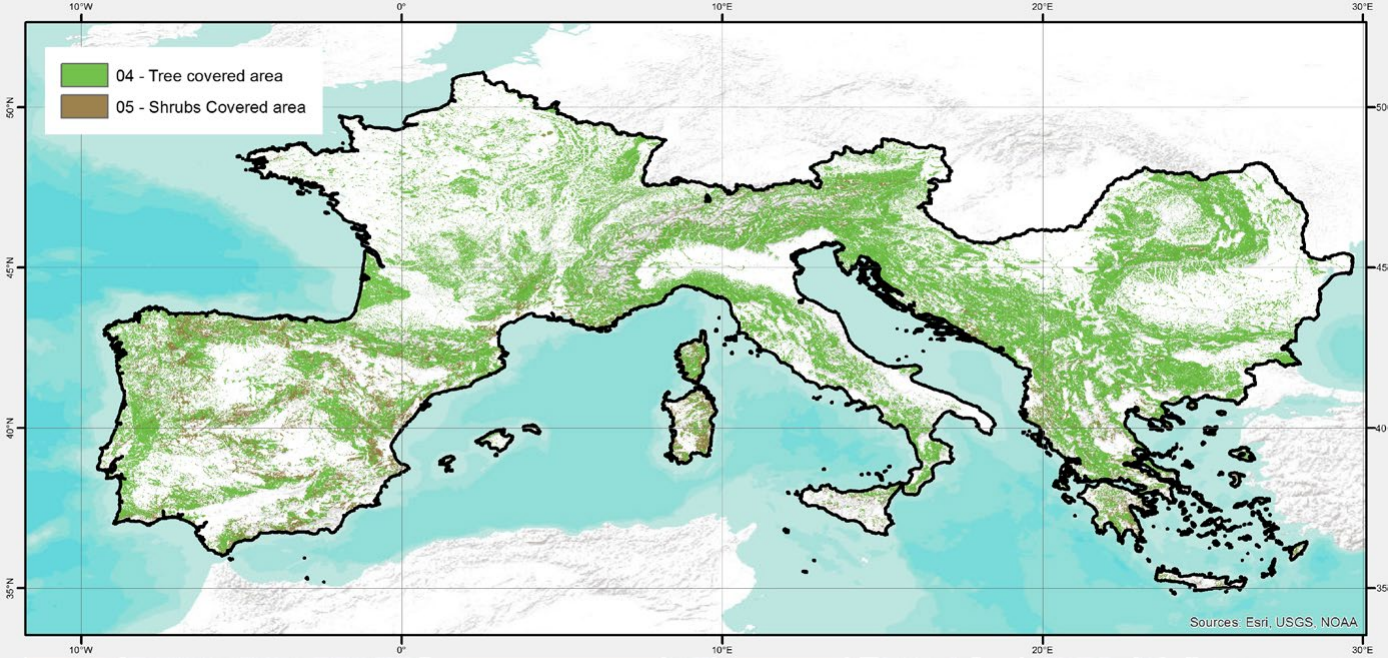
Envelope of study area (black boundaries) with forestlands and shrublands according to FAO Global Land Cover SHARE

The study area (Fig. 2) spreads from 10°W to 30°E longitude and from 24° to 50°N latitude. It covers the territories of 18 countries of southern Europe: Albania, Andorra, Austria, Bosnia and Herzegovina, Bulgaria, Croatia, France, Greece, Italy, Republic of Macedonia, Montenegro, Portugal, Romania, San Marino, Serbia, Slovenia, Spain, and Switzerland. The domain surface is approximately 2.34 million square kilometers, and about 30% of it is covered by forest (FAO, 2014). In turn, the 44% of forests is included into protected areas (protected planet^®^; www.protectedplanet.net).

According to CORINE Land Cover data for 2012 (http://land.copernicus.eu/pan-european/corine-land-cover/clc-2012/), 59% of the forestland domain is occupied by broadleaved species, 25% by coniferous, and 16% by mixed forests.

SDMs predict species occurrences in space and time based on simple to complex relationships between presence‐only or presence–absence point records of species and environmental variables. Aiming to explore the impacts of climate change on the geographic distribution of forest species in southern Europe, we investigated a large set of available spatial datasets on forest distribution (Trombik & Hlásny, 2013) and produced a database that expressed the fraction of presence for ten forest categories (i.e., grouping several species) as described in Noce et al. (2016). This database was created through forest category harmonization and spatial overlay procedures by merging two existing Pan‐European datasets: (1) the European Forest Institute (EFI) “Tree species maps for Europe” (Brus et al., 2012) and (2) the Joint Research Centre (JRC) “Novel Maps for Forest Tree Species in Europe” (Köble & Seufert, 2001). The forest category's distribution layers within this Source Database (hereafter SDb), originally at 30 arc‐sec resolution, have been upscaled to 0.25°, and the fractional presence value has been averaged within the new coarser cell by means of ESRI ArcGIS 10.1.

The 0.25° resolution was selected after several considerations. The most important one, as stated by Kriticos and Leriche (2010), is that maintaining a fine resolution without compatible resolution of climatic data leads to inaccurate projections and redundancy in data sampling. It should be taken into account that the original resolution of climate projections data by General Circulation Models (GCMs) from CMIP5 used here (see below) ranges from around 1° to 2.5° (see, e.g., Scoccimarro, Gualdi, Bellucci, Zampieri, & Navarra, 2013). Furthermore, we conducted a regional‐scale study and, as demonstrated by Guisan et al. (2007), very high resolution would not improve the accuracy of the SDMs. Conversely, too coarse resolution (approximately 0.5° or more) can identify inappropriate regions (Seo, Thorne, Hannah, & Thuiller, 2009). The adopted resolution was a compromise among the resolutions of the input datasets.

We subsequently created the Species Occurrence Database (SODb) by transforming continuous (percentage of presence) data in SDb into the presence/absence data as needed by SDMs. We assumed the values above 2% could be defined as “presence,” while lower values were considered as “absence.” This threshold allows excluding simulations of small and isolated populations that may represent relicts of past climate change (see Petit, Hampe, & Cheddadi, 2005) or small plantations. The ten forest categories considered in our work, with the associated (group of) species, are described in Table 1. Thus, SODb contained ten layers, each comprising 4,352 presence/absence locations (map units or pixels). Hereafter, the forest category name will be used instead of genus names.

**Table 1:** Name of forest categories and related species

Category name	Species
Abies	*Abies alba* Mill., *Abies cephalonica* Loudon
Betula	*Betula pendula* Roth, *Betula pubescens* Ehrhart
Castanea	*Castanea sativa* Mill.
Fagus	*Fagus sylvatica* L.
Larix	*Larix decidua* Mill.
Picea	*Picea abies* (L.) H.Karst.
PinusPin	*Pinus pinaster* Aiton
PinusSylv	*Pinus sylvestris* L.
QuercusRP	*Quercus robur* L., *Quercus petraea* (Mattuschka) Liebl.
QuercusSP	Other *Quercus* not included in QuercusRP

### 2.3 Environmental predictors and SDM calibration

Two categories of environmental predictors (EPs) have been considered for the calibration phase of SDMs. Nine (9) topographic predictors (TPs) and nineteen (19) bioclimatic predictors (BPs), as defined by WorldClim database, have been taken into account (Table 2; http://www.worldclim.org/bioclim). The WorldClim v.1.4 provides BP data for historical period (representative of 1960–1990; Hijmans, Cameron, Parra, Jones, & Jarvis, 2005), resampled up to 10 arc‐min (10′, approx. 0.16°) resolution. They were further upscaled in ESRI ArcGIS to 0.25° resolution with the help of bilinear method to fit the resolution of forest category presence data. Similarly, maps of TPs were produced starting from the Digital Elevation Model, as processed from the Shuttle Radar Topographic Mission data (SRTM; Farr et al., 2007) to create other topographic derivatives (slope, aspect) and descriptive statistics (mean, minimum, and maximum values). The 90‐m resolution of the original dataset was resampled to 0.25° resolution. Statistics for elevation and slope were calculated for the 90‐m resolution data within the new 0.25° pixel.

**Table 2:** Environmental predictors

Predictor ID	Description	Unit	Range (min–max)
Top1	Prevalent aspect	n.a.	1–10
Top2	Easting	degree	0–39.25
Top3	Latitude	degree	35.2–50.9
Top4	Max altitude	m	10–4,783
Top5	Max slope	degree	2.4–84.8
Top6	Mean altitude	m	3.5–2,730.4
Top7	Mean slope	degree	0.1–35.5
Top8	Min altitude	m	98–1,517
Top9	Min slope	degree	0–0.4
Bio1	Annual mean temp.	°C*10	−29–186
Bio2	Mean diurnal range	°C*10	49–129
Bio3	Isothermality	n.a.	20–47
Bio4	Temperature seasonality	°C*10	3,156–8,593
Bio5	Max temp of warmest month	°C*10	75–360
Bio6	Min temperature of coldest month	°C*10	−125–94
Bio7	Temperature annual range	°C*10	152–342
Bio8	Mean temp of wettest quarter	°C*10	−72–208
Bio9	Mean temp of driest quarter	°C*10	−84–266
Bio10	Mean temp of warmest quarter	°C*10	37–265
Bio11	Mean temp of coldest quarter	°C*10	−90–124
Bio12	Annual precipitation	mm	260–2,121
Bio13	Precipitation of wettest month	mm	35–238
Bio14	Precipitation of driest month	mm	0–153
Bio15	Precipitation seasonality	n.a.	7–100
Bio16	Precipitation of wettest quarter	mm	96–673
Bio17	Precipitation of driest quarter	mm	2–474
Bio18	Precipitation of warmest quarter	mm	2–556
Bio19	Precipitation of coldest quarter	mm	65–629

Several BPs can be highly correlated (or collinear), as they come from the same variables (temperature or precipitation) just operated at different scales (year, season, month), or even combined one another; however, the issue of whether or how treating collinearity in SDMs is still highly debated and unsolved (Dormann et al., 2013). First, collinearity is surely most important for linear models (De Veaux & Ungar, 1994) that are a very limited part of BIOMOD2 package mostly made of nonlinear and/or machine‐learning algorithms. Furthermore, collinearity is more noteworthy when the main goal is inferring the influence of predictors over occurrence, that is, for process understanding and/or interpretation purposes, while it seems not impacting predictions (De Veaux & Ungar, 1994). However, also in case of predictions, collinearity can be ignored only if the SDMs are applied over the same space and time, that is, if the collinearity structure remains constant (Dormann et al., 2013). While in our study the spatial domain is the same between calibration and projections, we also verified, by quantifying the collinearity among variables through the most common metrics (pairwise correlation coefficient, *r*, and the variance inflation factor, *VIF*; Naimi & Araújo, 2016), that the collinearity structure is not statistically different, at the 99.9% significance level according to the Wilcoxon signed‐rank test, across RCPs, GCMs, and time horizons.

Although mechanistic ecological knowledge for variable selection as well as more objective data reduction methods, such as principal component and factor analysis, can be used to reduce multicollinearity, this is often at the expense of interpretability (Miller, 2010).

Given all that above, we agree with Mateo, Felicísimo, Pottier, Guisan, and Muñoz (2012) that with no a priori reason for removing some variables, we can keep all of them for the analyses. The consequent risk of overfitting, likely for species with low number of occurrences, does not apply in our case, reducing the possibility of predicting more around the know presences (Mateo et al., 2012).

To train SDMs and test their predictive performances, we used a random subset of 70% points to calibrate the model for every single species/category, while the remaining 30% was used for the validation (see, e.g., Duque‐Lazo et al., 2016). We selected ROC and TSS as the evaluation metrics, because they are threshold independent and dependent, respectively, and could overcome the limits of Cohen's K. At the end, eight SDMs in BIOMOD2 were considered for the ensemble forecasting: GLM, GBM, CTA, ANN, SRE, MARS, RF, and MAXENT, all with the default settings (Phillips, Anderson, & Schapire, 2006; Thuiller et al., 2009).

For each forest category, models’ simulations that, after 10 runs, concurred to the ensemble were selected by applying a double statistic test (ROC and TSS) with a score of ≥0.85 (0.90 for QuercusRP and QuercusSP) for ROC and ≥0.75 for TSS, slightly enlarging the lower limits of good performance according to Zhang et al. (2015).

SDM ensemble projections in terms of probability of presence for each forest category were weightily averaged according to their performances and converted into a binary (presence/absence) prediction map using a threshold that maximized the evaluation statistics considered for this purpose, that is, ROC.

For these final maps, the analysis of performances was conducted via some metrics based on the confusion matrix.

### 2.4 Future projections and likelihood quantification

To project future forest categories’ distribution, the topographic predictors were considered unchanging, while for bioclimatic predictors we used those extracted by five bias‐corrected CMIP5 GCMs: GDFL‐CM3, HadGEM2‐CC, MIROC5, INM‐CM4.0, and CSSM4 (for acronyms and description see Scoccimarro et al., 2013) driven by RCP 4.5 and RCP 8.5. The RCP 4.5 is a stabilization scenario where total radiative forcing is stabilized, shortly after 2100, to 4.5 Wm^−2^ (approximately 650 ppm CO_2_‐equivalent) by employing technologies and strategies to reduce GHG emissions. The RCP 8.5 is a business as usual scenario and is characterized by increasing GHG emissions and high GHG concentration levels. It represents a rising radiating forcing pathway leading to 8.5 Wm^−2^ in 2100 (approximately 1,370 ppm CO_2_‐equivalent). Two time horizons were considered: 2050 (medium term) representing the 2041–2060 average and 2070 (long term) that represents the 2061–2080 average. The GCMs were selected as they cover a remarkable variability in terms of global future climate outlooks and, thus, provide a representative ensemble. Particularly, GDFL‐CM3 and INM‐CM4.0 are extreme in terms of annual mean temperature and precipitation amount (the former is the warmest and wettest; the latter is the coldest and driest). The other GCMs fall along an intermediate gradient. Moreover, GDFL‐CM3 projects a remarkable wetter world in the warmest quarter, while INM‐CM4.0 suggests global drier conditions in the coldest quarter.

All these data on future BPs were still downloaded from WorldClim database v.1.4 at 10′ resolution and resampled by means of ESRI ArcGIS 10.1 to 0.25°, adopting the bilinear method. Using these rescaled projections of BPs, we applied the previously calibrated SDM package of BIOMOD2 and produced ten *cascade ensemble members* (CEMs, each obtained by aggregating 8 SDM runs under 2 RCPs by 5 GCMs) for each time horizon and each forest category.

After producing maps of potential spatial distribution (namely climate‐driven habitat suitability, hereafter also simply “suitability”) of each forest category under each CEM, the spread in results due to GCMs and RCP scenarios was addressed by adapting the approach and terminology to treat the uncertainty and communicate the “likelihood” of outcomes, as proposed by IPCC‐AR5 (Mastrandrea et al., 2011). If the suitability is predicted only by 1 CEM, the outcome is considered as *extremely unlikely*; if by 2 or 3 CEMs, as *unlikely*; if by 4 to 6 CEMs, as *about as likely as not*; if by 7 or 8 CEMs, as *likely;* and if by 9 or 10 CEMs, as *extremely likely* (Table 3).

**Table 3:** Likelihood scale (based on IPCC AR5 guidance note Mastrandrea et al., 2011)

Term	Number of predicted suitability
Extremely unlikely	1
Unlikely	2–3
About as likely as not	4–6
Likely	7–8
Extremely likely	9–10

### 2.5 Changes in distribution and diversity

Two approaches were adopted to evaluate aggregated changes in terms of regional distribution and local diversity of forest categories, by averaging presence/absence results of the five GCMs for each RCPs and time horizons, called hereafter sub‐CEMs.

First, the re‐arrangement of forest categories’ frequency distribution was assumed a proxy of modifications in large‐scale forest composition (regional distribution) across the future new suitable areas for forests.

Second, the number of coexisting forest categories at spatial unit (pixel) level was considered representative of small‐scale forest composition (local diversity), and the updated frequency distribution of pixels with different presence (number) of forest categories was analyzed.

In both cases, the statistical significance of the differences in frequency distribution across RCPs and time horizons was assessed through a chi‐square test, at either full domain (regional) or biogeographical (subregional) level to distinguish subregions more or less vulnerable to diversity and distribution dynamics. Biogeographical regions considered are those covering currently 95% of the study domain (Atlantic, Alpine, Continental, Mediterranean), and their extent was taken from the European Environment Agency 2015 dataset (http://www.eea.europa.eu/data-and-maps/data/biogeographical-regions-europe-3).

By combing the two approaches, it is possible to evaluate changes in both large‐scale distribution and local diversity seeing, for example, if the general forested areas increase/decrease but the large‐scale composition remains unaltered, or if the diversity of the spatial units remains “quantitatively” similar under future outlooks but it is due to a mosaic of different forest categories.

## 3 RESULTS

### 3.1 Ensemble SDM performance and predictors’ importance

As described in Miller (2010), there are several measures to test the predictive performance of SDMs. Table 4 shows results in terms of Sensitivity (Sens), Specificity (Spec), Predicted Present correctly Predicted (PPP), Predicted Absent correctly Predicted (NPP), True Skill Statistic (TSS), Percent Correctly Classified or Overall Accuracy (PCC), Cohen's Kappa, and Matthews index (MCC). Average performances range from 0.807 for Fagus to 0.966 for Castanea, and they can be considered from good to excellent if assuming the classification of Zhang et al. (2015).

**Table 4:** Binary Models accuracy. Sensitivity (Sens), Specificity (Spec), Predicted Present correctly Predicted (PPP), Predicted Absent correctly Predicted (NPP), True Skill Statistic (TSS), Percent Correctly Classified or Overall Accuracy (PCC), Cohen's K, and Matthews index (MCC)

Category	Sens	Spec	PPP	NPP	TSS	PCC	Kappa	MCC	Average
Abies	0.968	0.973	0.916	0.990	0.942	0.972	0.923	0.924	0.951
Betula	0.974	0.979	0.933	0.992	0.953	0.978	0.939	0.939	0.961
Castanea	0.977	0.979	0.950	0.990	0.956	0.978	0.948	0.948	0.966
Fagus	0.814	0.919	0.938	0.766	0.733	0.856	0.709	0.718	0.807
Larix	0.994	0.981	0.879	0.999	0.975	0.983	0.923	0.926	0.958
Picea	0.983	0.952	0.887	0.993	0.934	0.960	0.904	0.907	0.940
PinusPin	0.974	0.947	0.802	0.994	0.922	0.952	0.850	0.856	0.912
Pinus Sylv	0.979	0.972	0.962	0.985	0.951	0.975	0.948	0.949	0.965
QuercusRP	0.979	0.969	0.975	0.973	0.948	0.975	0.948	0.948	0.964
QuercusSP	0.973	0.963	0.970	0.967	0.937	0.969	0.937	0.937	0.957

Table 5 shows the standardized importance of environmental predictors. Given also the collinearity issue mentioned in Methods’ section, this importance can be analyzed mainly looking at group of predictors, as they are often based on the elaboration of the same variable (precipitation or temperatures) at different temporal scales, or they come from a further combination of predictors.

**Table 5:** Standardized predictors importance (average among SDMs)

Variable	Abies	Betula	Castanea	Fagus	Larix	Picea	Pinus Pin	Pinus Sylv	Quercus RP	Quercus SP	Avg	*SD*
Bio1	4.813	4.336	5.094	4.007	3.581	4.012	4.391	7.103	3.554	7.532	4.842	1.323
Bio2	2.761	1.485	1.997	2.035	1.271	1.800	2.569	1.811	3.126	1.502	2.036	0.573
Bio3	2.058	1.710	0.662	1.681	0.818	0.817	1.405	2.373	0.522	1.054	1.310	0.599
Bio4	3.655	5.935	2.829	5.136	3.566	3.088	5.003	5.264	4.469	6.922	4.587	1.244
Bio5	4.226	2.056	1.586	2.992	2.241	2.411	2.523	5.779	3.678	9.877	3.737	2.358
Bio6	4.160	1.535	7.221	2.120	3.704	2.831	3.375	5.708	3.298	4.553	3.850	1.592
Bio7	5.362	2.506	2.256	2.536	1.856	3.213	2.418	3.300	4.542	4.191	3.218	1.081
Bio8	0.742	0.941	0.865	0.872	0.932	0.687	3.129	1.798	1.268	0.629	1.186	0.724
Bio9	1.469	2.979	1.301	1.738	1.998	2.413	6.160	2.332	2.230	1.637	2.426	1.333
Bio10	3.464	10.624	4.614	4.712	4.542	3.142	4.789	4.465	9.239	5.153	5.474	2.323
Bio11	7.221	5.686	6.460	8.726	4.561	3.745	4.059	8.012	6.704	7.046	6.222	1.591
Bio12	8.198	9.165	17.318	7.020	15.790	7.174	6.983	3.768	9.178	3.739	8.833	4.267
Bio13	3.066	3.725	2.312	1.337	3.725	4.106	2.601	3.126	1.969	1.431	2.740	0.924
Bio14	9.745	10.132	3.192	7.669	5.401	1.789	3.123	1.933	8.759	1.773	5.351	3.255
Bio15	4.165	5.675	7.350	2.583	4.639	2.123	3.521	5.702	1.685	1.643	3.909	1.842
Bio16	2.921	3.635	4.754	2.278	4.867	3.085	4.219	3.804	5.939	3.404	3.890	1.025
Bio17	4.194	5.708	7.164	3.844	3.520	4.186	3.394	5.128	4.309	3.890	4.534	1.102
Bio18	4.192	5.307	5.195	5.836	10.073	22.406	8.746	4.126	4.738	11.396	8.201	5.324
Bio19	3.189	3.515	4.211	2.892	3.936	2.678	5.429	2.095	2.381	4.245	3.457	0.966
Top1	0.044	0.072	0.076	0.044	0.038	0.069	0.042	0.045	0.047	0.040	0.052	0.014
Top2	5.367	2.356	2.152	7.988	3.955	4.498	10.942	3.156	5.001	2.122	4.754	2.681
Top3	1.195	1.998	2.653	2.397	3.499	8.577	1.891	4.117	3.238	9.992	3.956	2.800
Top4	5.897	3.058	1.422	7.980	5.296	2.967	2.577	3.894	1.482	2.108	3.677	2.024
Top5	2.081	1.125	0.695	2.512	1.162	1.005	1.299	2.202	0.862	0.994	1.394	0.600
Top6	2.542	2.426	1.561	6.043	3.045	4.447	2.509	3.043	4.872	1.177	3.166	1.440
Top7	1.348	0.624	1.147	0.863	0.976	0.866	1.014	1.075	0.752	0.889	0.956	0.120
Top8	1.816	1.635	3.851	2.129	0.980	1.828	1.792	4.754	2.029	0.958	2.177	1.143
Top9	0.018	0.052	0.063	0.028	0.028	0.039	0.098	0.088	0.125	0.104	0.064	0.035
Variance	5.436	8.074	12.123	6.465	10.169	17.277	5.986	3.775	6.813	10.199	—	—

An overview of all forest categories demonstrates that Bio12 (annual precipitation), Bio18 (warmest quarter precipitation), Bio11 (winter mean temperature), and Bio14 (driest month precipitation) present the highest incidence.

The single most influencing predictor is Bio18 for Picea (22.36%). Notably, Bio12 appears crucial for all categories, except PinusSylv and QuercusSP. QuercusRP and Betula are sensible to Bio10 (summer mean temperature), showing importance of 11.2% and 11.9%, respectively. Among topographic predictors, Top 2, 3, and 4 (representing easting, latitude, and the peaks of altitude and being not correlated one another) prove to have larger effect on model calculations, and this could reflect upon the most inland/continental conditions of the domain. Instead, Top 1, 7, and 9 (related to prevalent aspect and the mean and minimum slope, not correlated) appear totally marginal. Examining the variance shows that, for some species, the importance is concentrated in few predictors (e.g., Castanea, Larix, or Picea). However, for Abies, Fagus, PinusPin, and PinusSylv in particular, no noticeable difference is detected among predictors.

Moreover, we must consider that the analyzed importance is an average among SDMs and thus can differ across them. Some SDMs can also remove predictors from the analysis so that the predictor importance is null in that case. In fact (results not shown), when considering all the forest categories together, the SRE model (see methods) always keep all the predictors, similarly occurs for RF model, except for the predictor Top 1 (prevalent aspect) and Top 9 (minimum slope) that rarely have an importance also for other models. The most selective model seems MARS, where only ten predictors are kept for more than 70% of runs. However, BPs seem in general more important than TPs, as they are held in the SDMs for 84% and 70% of runs, respectively. Finally, on average across forest categories, the predictors kept most frequently (>90% of runs) appear those related to temperature (annual mean, seasonality, diurnal range, wettest season) and geographic location (easting and latitude).

### 3.2 Future projections

For each forest category, 10 maps (5 GCM by 2 RPCs) were produced and then aggregated into a final map (one for each time horizon) that shows the likelihood of outcomes according to IPCC terminology. Figs 3 and 4 (for Fagus and QuercusSP) are reported in this study as examples. All other maps are available in the Supporting Information (Figs. S1–S16). Gray areas show historical presence, while red tones demonstrate the degree of likelihood for future climate‐driven habitat suitability.

**Figure 3:**
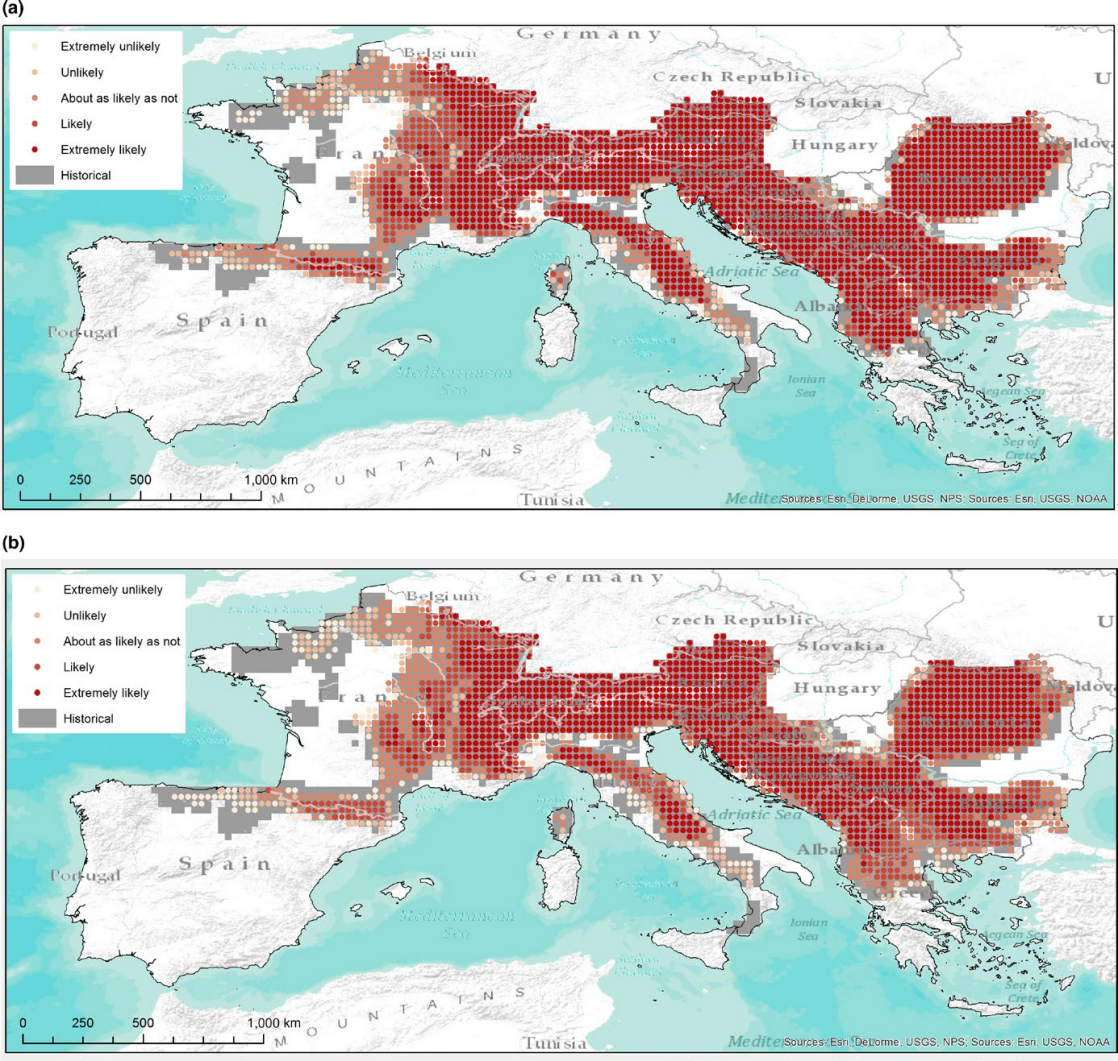
Future likelihood suitability map for Fagus: 2050 (a) and 2070 (b)

**Figure 4:**
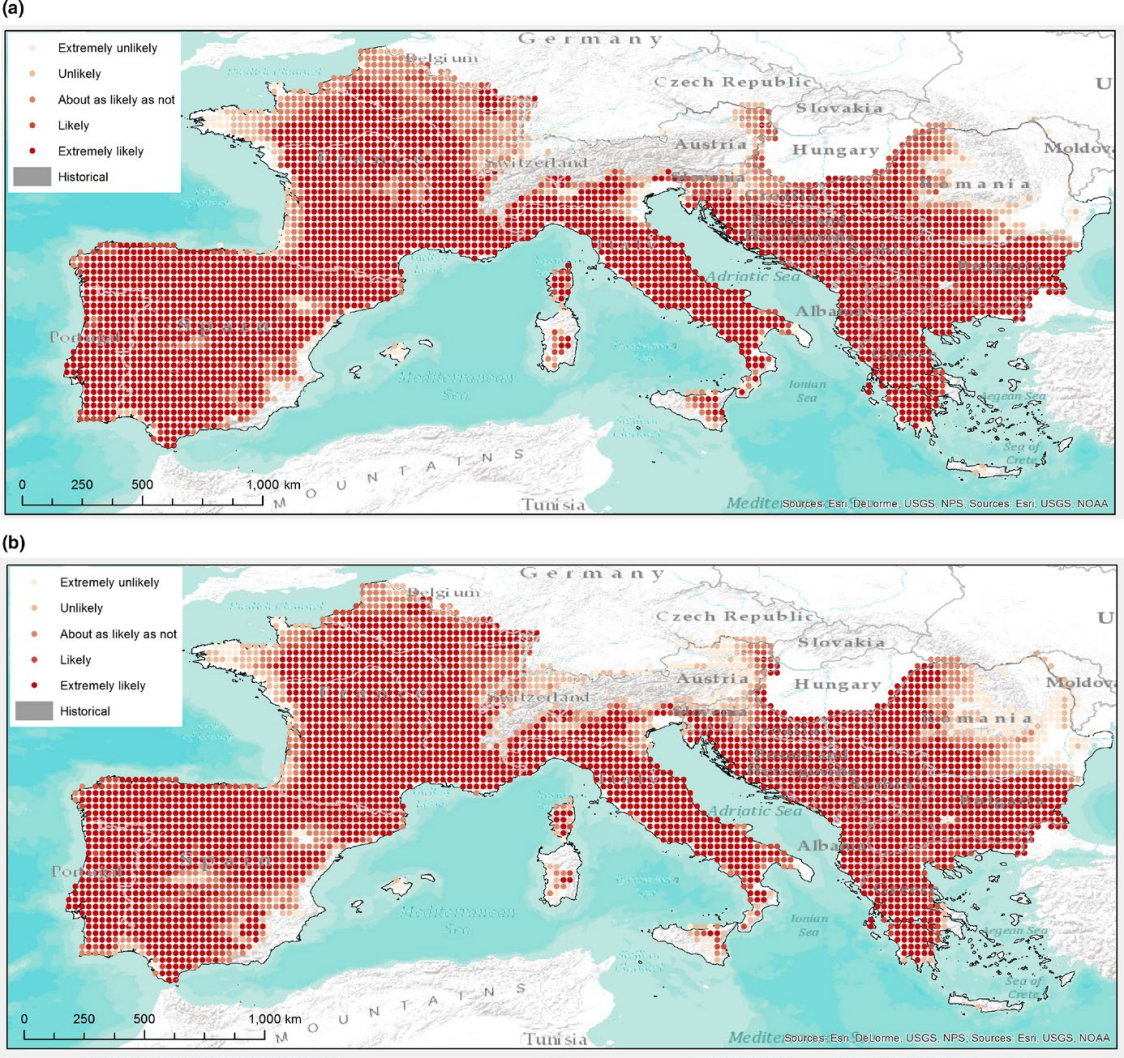
Future likelihood suitability map for QuercusSP: 2050 (a) and 2070 (b)

**Figure 5:**
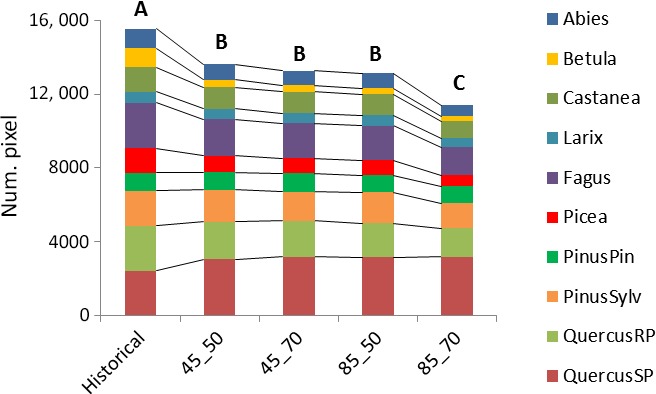
Changes in forest distribution in number of pixels. Different capital letters mean that distributions are significantly different at the 99.9% level (*p*‐value < .001)

Concerning the changes in large‐scale distribution and diversity, Fig. 5 shows that, in general, the whole domain is expected to suffer from a reduced suitability for the forest categories analyzed. All sub‐CEMs’ projections (see also Figs. S17–S20) are significantly different from historical conditions in terms of categories distribution, with the main change being the reduction of Betula (with respect to the whole historical distribution), Picea (upward altitudinal shifting), Castanea (France and Italy), Fagus (France, Northern Spain, Central, and Southern Italy), Larix (partially balanced by new suitable areas in the Dinaric Alps), QuercusRP (France mainly and Spain also, partially balanced with an increase on the Central Alps), and Abies (reduction in the Western edge balanced by increasing suitability in the Dinaric Alps and the Carpathians) these against a spread of QuercusSP in the northern side of study area (Fig. 4) and of PinusPin. PinusSylv deserves attention, as suitability seems to be guaranteed over time (with a gain in Romania and Northern France) but, especially in the long term, with a low level of likelihood.

Medium‐term projections under both RCPs do not differ significantly in terms of forest category distribution. Besides, the RCP 4.5 is similar to the RCP 8.5 in the medium term. Generally, the dynamics of future changes identified in the medium term are emphasized in the long term.

**Figure 6:**
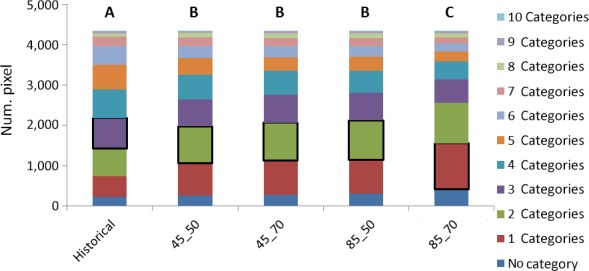
Changes in forest diversity. Bordered portions of bars represent the most frequent combination of categories. Different capital letters mean that distributions are significantly different at the 99.9% level (*p*‐value < .001)

The long‐term sub‐CEMs under RCP8.5 have significantly different distribution with respect to both historical conditions and the medium term or RCP4.5 projections. In this reduced forest occurrence, the local diversity seems to be lost at statistically significant level, as the currently most frequent mix of three forest categories is substituted by the highest frequency of map units made of two categories (medium term or RCP4.5) up to one category alone in the long‐term RCP8.5 (Fig. 6).

Looking at biogeographical region level, some peculiarities emerge, with the Atlantic (Fig. S21), Continental (Fig. S22), and Mediterranean (Fig. S23) regions suffering from large losses and significant modifications in forest distribution and diversity.

For the Atlantic subregion, the main modification is determined by the reduction of suitability for Betula, Castanea, Fagus, QuercusRP, against a spread of QuercusSP and PinusPin. While in the historical period, the most frequent combination is made by four forest categories, which are present in the same map unit, in the long‐term RCP8.5 most of forested pixels are composed of one class, after a dominance of units with two forest categories under either RCP4.5 or medium‐term sub‐CEMs.

The Continental region is similar to the full domain for that concerning the significance of the differences among time horizons and RCPs, with the long‐term RCP8.5 significantly diverging from the other projections. Initially, a spread of QuercusSP is projected that reaches a suitability comparable to the dominant Fagus; when the long‐term RCP8.5 conditions occur, the prevalence reverses, and there is also a progressive reduction for the other oaks analyzed (QuercusRP). The highly diversified landscape with most of map units hosting five forest categories is expected to experience the loss of local diversity under all projections, where four categories in the same pixel prevail even if in the long‐term RCP8.5 scenario, the areas with two and three categories become very frequent.

The Mediterranean region presents the most affected trends in either long term or RCP8.5 sub‐CEMs, significantly different from those under medium term or RCP4.5 sub‐CEMs, and especially from historical conditions. This fact suggests accelerated dynamics of suitability loss for the considered forest categories and a re‐arrangement in distribution. The region is projected at risk of progressive disappearing of Betula, Larix and Picea; a reduction of more than a half of Fagus, PinusSylv and QuercusRP; a weaker reduction in Abies, Castanea and PinusPin; and a confirmed dominance of QuercusSP. The smoothly diversified landscape, which appears largely represented by map units with two forest categories, loses diversity, and the zones with a single forest class seem dominating under all future projections.

A diverging trend is shown by the Alpine region (Fig. S24). After a slight overall increase of forest presence under either RCP4.5 or medium‐term sub‐CEMs, with significant re‐arrangements of landscape forest composition, an even slighter reduction is projected toward the re‐establishment of the “historical” presence (but with a noticeable reduction in Betula and Picea) and a spread of the two Quercus categories, although the dominance of Fagus seems to be maintained. In terms of local diversity, no significant difference exists across sub‐CEMs, suggesting that, under this preserved “quantitative” diversity, new species seem finding suitable conditions in the Alpine areas.

## 4 DISCUSSION

The findings of this work should be regarded as “habitat suitability” or simply “suitability,” referring to favorable habitats for the analyzed forest categories rather than to their expected future spatial distribution under the impacts of climate change, because of three main points.

First of all, the investigated forest categories represent a significant portion of species in Southern Europe but, obviously, they neither cover the totality of current forest diversity, nor are representative of other species in the adjacent regions that can migrate toward southern Europe. Thus, competitive dynamics (expressed here as higher or lower probability of occurrence among categories) should also consider other species not included in this study.

Similarly, the other land uses/covers (agriculture, artificial surfaces, water bodies), well established in the domain, are not considered in our analysis, neither in their current locations nor in the likely new areas of expansion.

Furthermore, to interpret the suitability directly as an indicator of forest presence/absence, species migration should be considered free from ties, and constraining peculiarities of the study area should not be taken into account. Such peculiarities comprise the high degree of fragmentation of habitats owing to intensive anthropic land use, including the dense network of infrastructure barriers, and the topographic limits related to high topographic heterogeneity (Saltré, Duputié, Gaucherel, & Chuine, 2015). In this regard, the capacity of individual species to colonize new favorable areas or to adapt in existing ones depends partially on their competitive and dispersal capabilities (Boulangeat, Gravel, & Thuiller, 2012; Corlett & Westcott, 2013; Schiffers, Bourne, Lavergne, Thuiller, & Travis, 2013; Zhu, Woodall, & Clark, 2012).

Moreover, the identified likely transitions from a forest category to another are “potential” and we can imagine they cannot take place by natural succession in a short timeframe like the next 50–60 years, as expansion rates of species are much slower than the expected rate of warming: to bridge 100 km, an acorn‐bearing species like oaks or beech may need 500 years or even more (Saltré et al., 2013), while wind‐distributed tree species, like birch or some conifers, may reach much faster expansion rates. For this reason, the transition to a new equilibrium has to be supposed including ecological imbalances, like the appearance of unstable communities dominated by wind‐propagated, pioneer tree species or even intermittent deforestation of wide landscapes (Bussotti, Pollastrini, Holland, & Brüggemann, 2015). Although climatic changes are still too rapid to allow a natural rebalancing of forest ecosystems (Corlett & Westcott, 2013), given the time horizons of this work, the species migration dynamics are considered more facilitated to adapt to new climatic conditions than evolutionary ones (Wiens, 2011).

However, the produced maps of future suitability allow summarizing three kinds of impacts on the potential re‐distribution of forest categories under alternative climate outlooks:

- *Minor or no impacts on the historical distribution while new areas become suitable*. PinusPin and QuercusSP belong to this group, as they seem to be positively affected by changing conditions, and the expected increasing temperature in particular (Giorgi & Lionello, 2008). PinusPin gains favorable conditions in some areas in which it is not present nowadays (e.g., Northern France, the Western Alps, and the Pyrenees, although with high level of uncertainty). QuercusSP's suitability future spreading appears extremely wide in both northward and eastward directions (the whole France, except Bretagne, and Western Romania). Similar results concerning QuercusSP are presented in Zimmermann, Normand, Pearman, and Psomas (2013) and Spathelf, Van Der Maaten, Van Der Maaten‐Theunissen, Campioli, and Dobrowolska (2014).
- *Negative impacts on some portions of historical distribution and, in some cases, with suitability gain for others*. Long‐term projections of suitability for Abies show its potential disappearing in France as well as in the Apennine populations; however, suitability can increase across the Dinaric Alps and the Carpathians, suggesting an eastward shifting of the range. Both medium‐ and long‐term projections for Larix show a good capacity of adaptation to changing conditions in historical presence zones, and a slightly increasing suitability in new areas to be potentially colonized. More noticeable could be the impacts on QuercusRP and the western side of its range in particular; this scenario, only partly compensated with increasing suitability in Romania, can have important economic repercussions (Hanewinkel, Cullmann, Schelhaas, Nabuurs, & Zimmermann, 2012), considering the importance of the oak timber market in France. Projections indicate that PinusSyl retains a medium suitability throughout its range, although new areas of possible colonization in the eastern portion of the study area are detected. Finally, for Fagus, projections show that suitability will be seriously threatened, decreasing in great part of western and southern edges of its historical distribution (clear and alarming example in the Central and Southern Apennines). In this case, as for Abies, there is evidence of eastward shifting of the range. These results are in accordance with other studies (Falk & Hempelmann, 2013; Hickler et al., 2012; Kramer et al., 2010; Saltré et al., 2015).
- *Negative impacts on the most part of historical distribution*. According to our results, extremely likely and likely suitability for Betula will be guaranteed only in few areas (the Western, Central, and Dinaric Alps) with a strong reduction in the remaining range (particularly in France). Likewise, especially long‐term impacts on Castanea are significant and largely widespread, matching with the results of Goberville et al. (2015). Particularly, suitability sensibly decreases in areas (Southern Italy), which are already affected by serious diseases (Vettraino et al., 2009). Picea's distribution could be negatively impacted on the medium and long term in the peripheral region of the range; the upward altitudinal shifting matches with Falk and Hempelmann's (2013) study.

The above reported regional changes in distribution match with results of Hanewinkel et al. (2012), even if under previous IPCC scenarios, for that concerning the general contraction of Picea, Fagus, Mediterranean oaks (Quercus SP), PinusSylv and PinusPin, while for Quercus RP, their projections suggest an expansion but this is related to the fact that their study area cover the whole Europe while in our work any potential shift is constrained toward the north‐eastern margins of the domain. These results lead to think also in terms of economic consequences: Hanewinkel et al. (2012) concluded that based on their results, the expected value of European forest land will decrease owing to the decline, in the absence of effective countermeasures, of economically valuable species replaced by lower‐value oak communities. These results are confirmed by other studies (Hickler et al., 2012; Lindner et al., 2014; Resco de Dios, Fischer, & Colinas, 2006; Santini et al., 2014), and they are in line with projected expansion toward the north of the Mediterranean‐like climate (Alessandri et al., 2014).

Our results need to be also considered with respect to the species auto‐ecological traits. Considering that climate change projections over the study area foresee increasing summer droughts, our results substantially confirm, in terms of future suitability changes, some well assessed climate dependencies of forest species. *Abies alba* Mill., *Fagus sylvatica* L., *Betula* sp., *Quercus robur* L., and *Picea abies* (L.) H.Karst.*,* indeed, are all rain‐demanding forest species and in particular exigent of summer precipitation (Bernetti, 2005); thus, their contraction could be explained by their high sensitivity to this bioclimatic variable (*i.e.,* Bio18) as already seen in further studies (Boden, Kahle, Wilpert, & Spiecker, 2014; Macias, Andreu, Bosch, Camarero, & Gutiérrez, 2006; van der Werf, Sass‐Klaassen, & Mohren, 2007; Zang, Pretzsch, & Rothe, 2012; Zimmermann, Hauck, Dulamsuren, & Leuschner, 2015).

Meanwhile, the expected spreading of QuercuSP can be explained by the variety and the heterogeneity of this categories, but special attention must be paid to the auto‐ecological traits of the species within; in particular the high resilience of these Mediterranean species to drier and warmer conditions (e.g., *Quercus ilex* L., *Quercus pubescens* Wild, and *Quercus cerris* L.) as described in Baldocchi et al. (2010), Morán‐López, Poyatos, Llorens, and Sabate (2014), and Vaz et al. (2010).

Recent studies regarding a mountain area in Slovakia (Hlásny et al., 2017) reported the crucial importance of annual temperature and of temperature in the warmest and coldest months for productivity of some *Abies* species as reported also by Lebourgeois (2007) and Carrer, Motta, and Nola (2012) for the Alpine chain; while recognizing the role of the coldest period's temperature, our results seem more influenced by other predictors based on precipitation (i.e., annual and of the driest month). These discrepancies could be related to the different attributes of study areas in terms of climatic heterogeneity and geographical extent it is reasonable to presume that some climatic variables have a different importance in different contexts but also can reveal a different influence according to the whole set of variables they belong to. Despite the results of the predictors’ importance analysis, annual precipitation would not play a predominant role in future changes, especially in the northern‐eastern boundaries of our domain.

The differences in changing large‐scale forest composition highlighted across bio‐geographical regions can reinforce further studies, as well as corroborate the existing ones, related to the assessment of impacts due to extreme events and to either biotic or abiotic disturbances on the forest status and carbon cycle. For example, Seidl, Schelhaas, Rammer, and Verkerk (2014) assessed how, under current forest distribution, growth dynamics and management, the different eco‐regions are affected by different disturbances, ranging from fires in the south to bark beetles and wind in the north. Those same authors project the opportunity for improving carbon stock from European forests by changing management, under combination of climate change and disturbances, but not considering potential changes in forest habitats and thus new landscape composition.

Local scale diversity, analyzed in terms of potential presence in the same map unit, shows an appreciable reduction, especially on the long term, throughout the study domain. This alarming trend affects almost all bio‐geographical subregions as described in other studies (Sala et al., 2000; Thuiller, Lavorel, Araujo, Sykes, & Prentice, 2005). The Alpine subregion, nevertheless, seems to be less affected, perhaps due to the combined effect of reduction of mountainous species (e.g., *Picea abies*) and new colonization of mesophilic species (*Quercus robur* and *Q. petraea*) that might find more suitable climatic conditions. Similar effects on alpine vascular vegetation have also been described by Pauli, Gottfried, Reiter, Klettner, and Grabherr (2007). Although observed altitudinal shift in the Swiss Alps have been associated more to land abandonment rather than to climate change (Gehrig‐Fasel, Guisan, & Zimmermann, 2007), our study considers potential suitability, so the space left or not by other land uses does not influence the outcomes. The topic of diversity, even limited to the (group of) species level, is of strategic importance to increase ecosystem productivity because resources are better shared among neighboring species and are thus potentially more available (Loreau et al., 2001). Moreover, Jactel and Brockerhoff (2007), Lebourgeois, Gomez, Pinto, and Mérian (2013), Pretzsch, Schütze, and Uhl (2013) and Zhu et al. (2000) demonstrated that diversity increase resilience to both biotic stresses such as insect pests or diseases and abiotic stresses as droughts, especially for drought‐prone areas as southern Europe. This point is crucial given the expected increase of drought frequency for the Mediterranean area (Vicente‐Serrano et al., 2014).

## 5 CONCLUSION

In this work, we coupled a set of SDMs with alternative climate projections under different global circulation models and emission scenarios. We aimed first at investigating the likelihood of future changes in the climate‐driven habitat suitability, or simply “suitability,” including the modification in (regional) distribution and (local) diversity, for some forest categories in Southern Europe. Then, the *cascade ensemble system*, allowed to treat together both SDMs and climatic projections’ uncertainty, providing probabilistic and thus more robust information about potential future changes in forest habitats under changed environmental conditions.

Results in terms of forest shift, regional composition, and local diversity confirmed how climate change is likely to led to significant modifications in the future, affecting forests with different degrees of magnitude across species and with various levels of uncertainty within species also due to the spatial heterogeneity, the auto‐ecological traits, and the adaptation strategies.

While some forest categories will find more suitable conditions in previously unsuitable locations, for other categories, the same new conditions will become less suited. A decrease in local species diversity is projected in most of the area, with Alpine region showing the potentiality to become a refuge for species migration.

Results, flagged with the related likelihood, represent useful and immediate information to be communicated to stakeholders and policy makers for supporting their design of future forest conservation as well as protection strategies and plans.

## CONFLICT OF INTEREST

The authors declare that they have no conflict of interests.

## AUTHOR CONTRIBUTIONS

S.N. and M.S. conceived the idea; S.N performed all the analyses with GIS and Biomod2 package and led the writing; M.S. conducted the spatially aggregated diversity and distribution analyses; and A.C. supported in the evaluation of predictors importance. All authors actively contributed to the discussion of results.
