# Supplementary material for: Likelihood of changes in forest species suitability, distribution, and diversity under future climate: The case of Southern Europe
Source: Ecol Evol. 2017 Oct 7;7(22):9358–75. doi: 10.1002/ece3.3427 (PMC5696419; doi:10.1002/ece3.3427)
Supplement: Supplementary file 1 [file ECE3-7-9358-s001.docx]

**SUPPORTING INFORMATION1**

**Supporting Information 1**

**Appendix S1** Future likelihood suitability maps at mid (2050) and long (2070) term for Abies, Betula, Castanea, Larix, Picea, PinusPin, PinusSylv, QuercusRP.

**Figure S1** Future likelihood suitability map for Abies (2050)


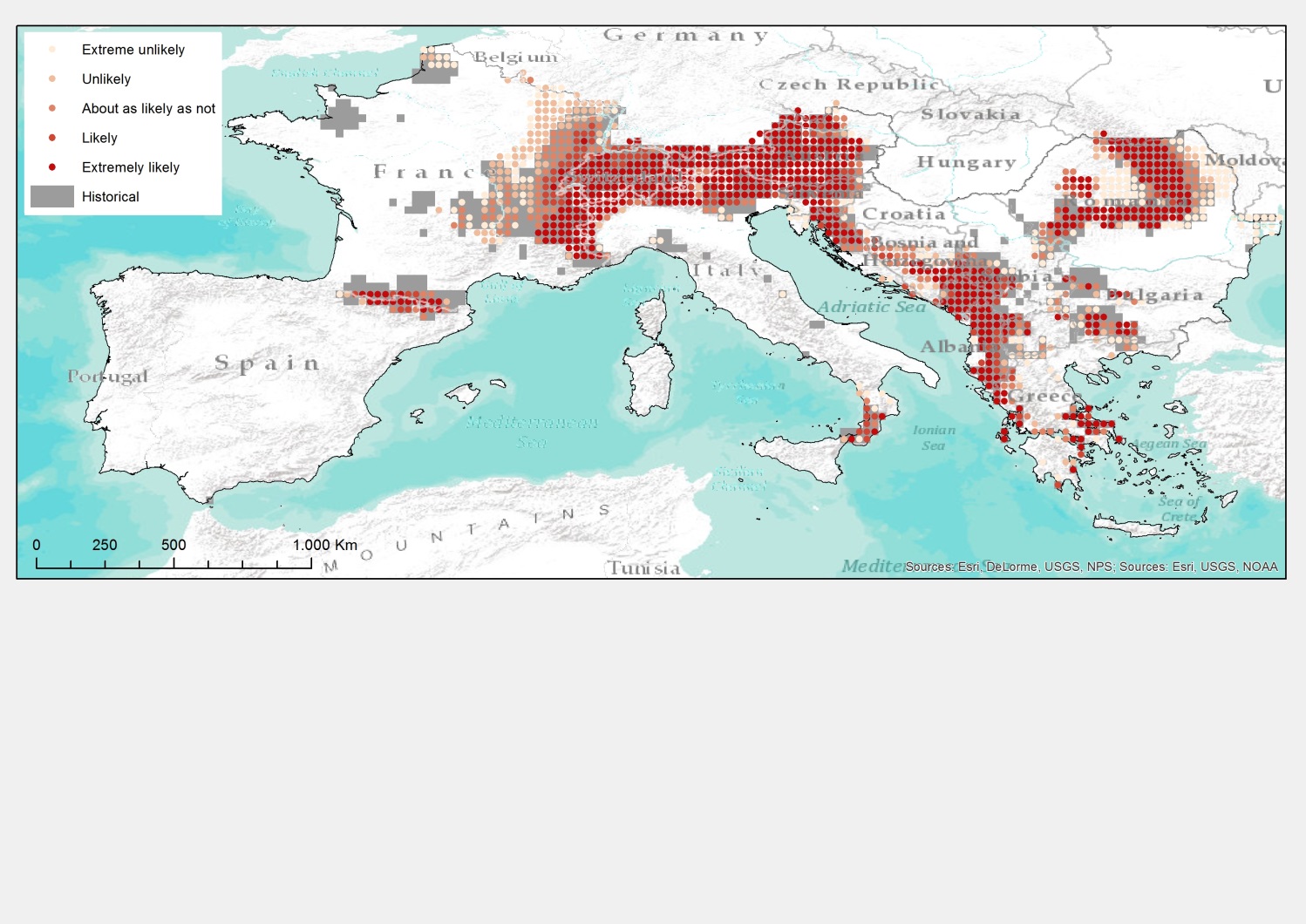


**Figure S2** Future likelihood suitability map for Abies (2070)


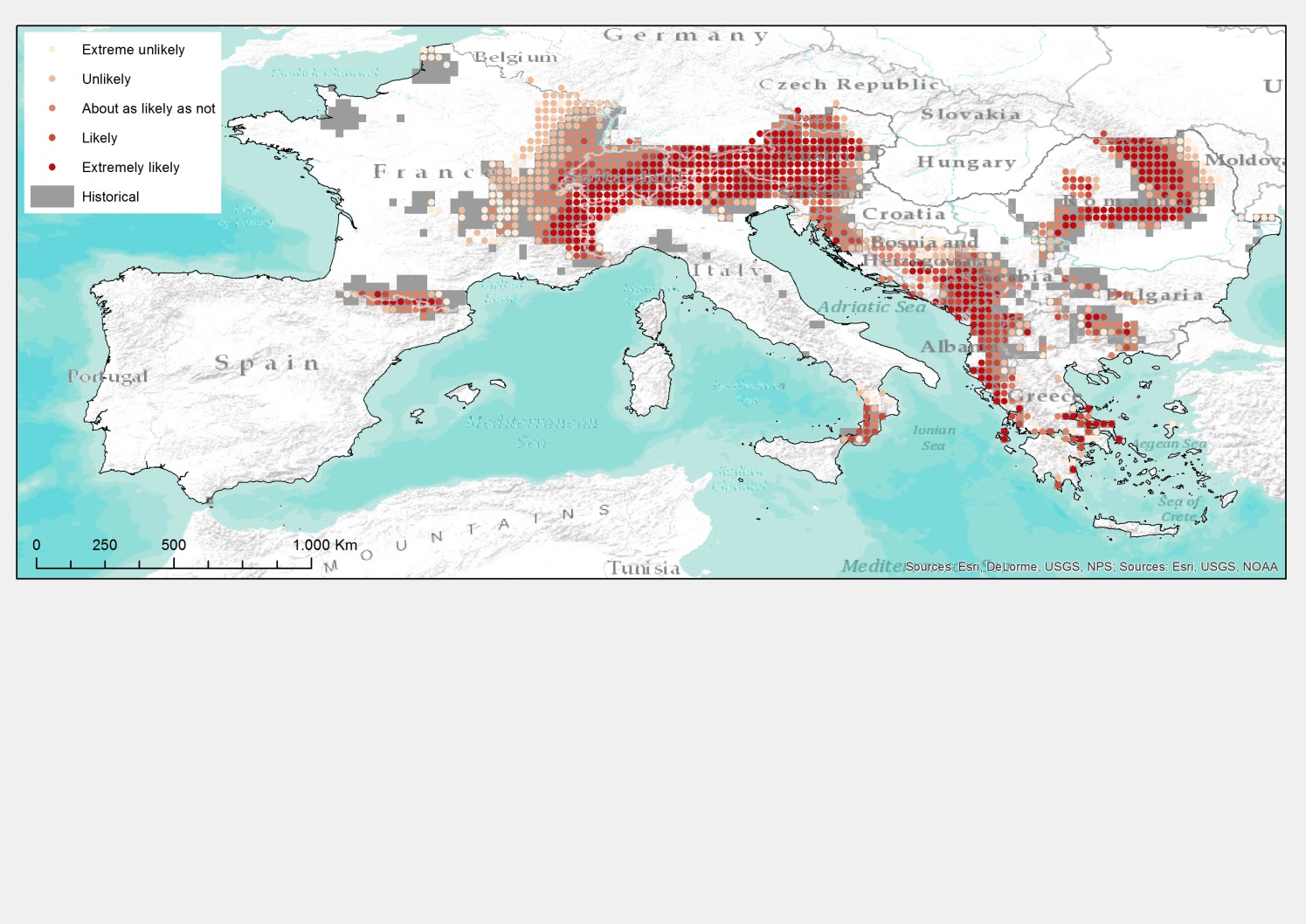


**Figure S3** Future likelihood suitability map for Betula (2050)


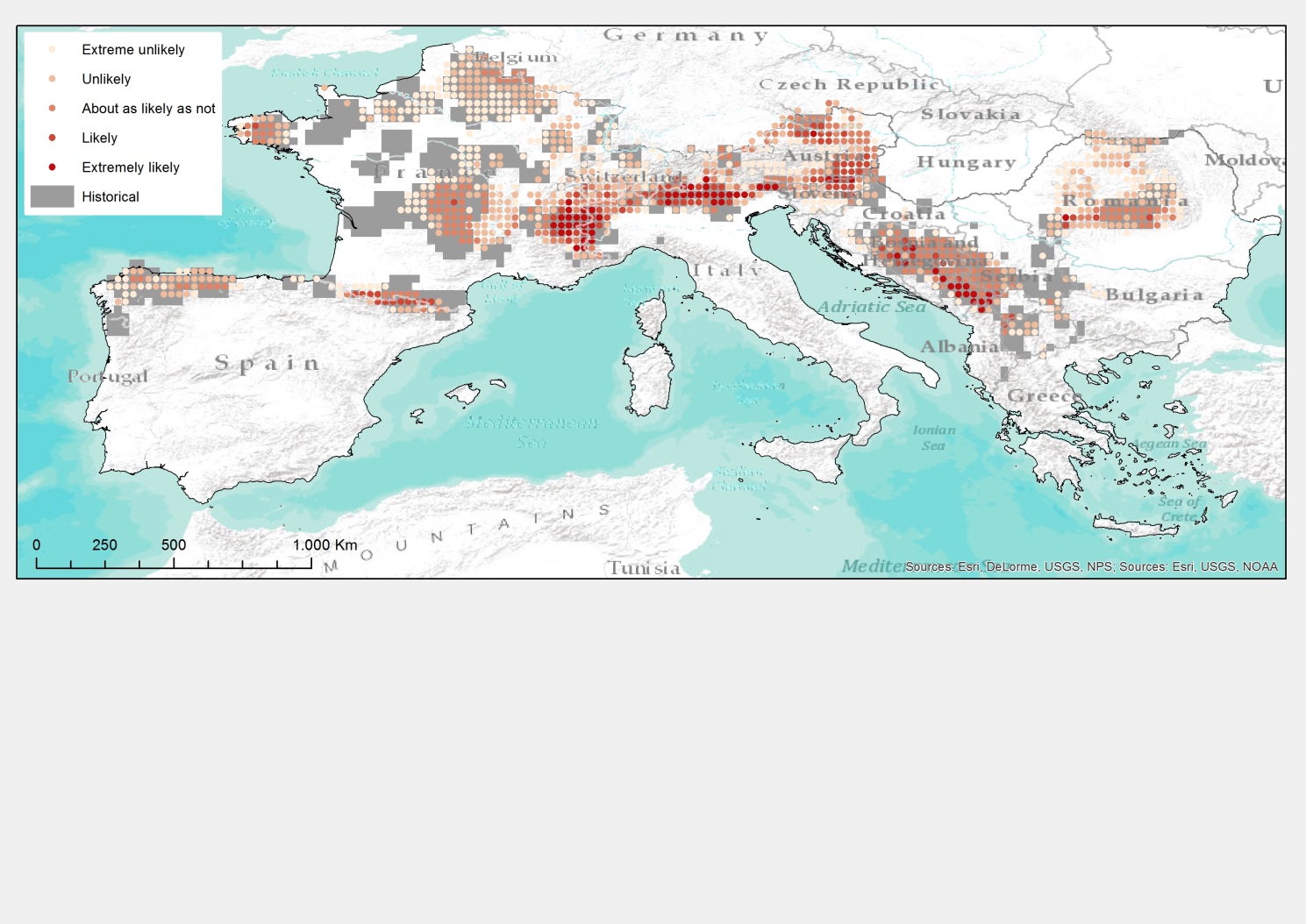


**Figure S4** Future likelihood suitability map for Betula (2070)

**
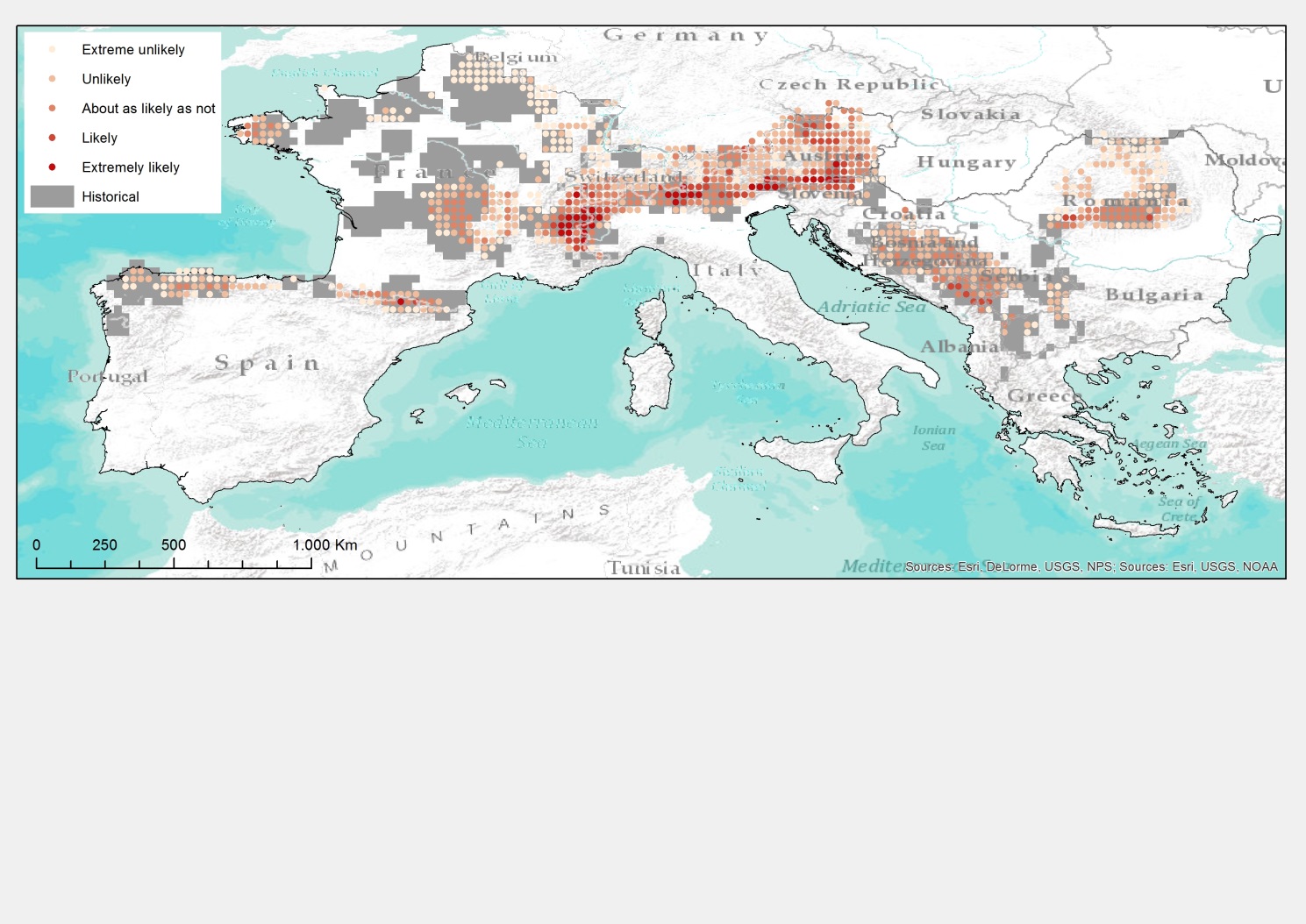
**

**Figure S5** Future likelihood suitability map for Castanea (2050)

**
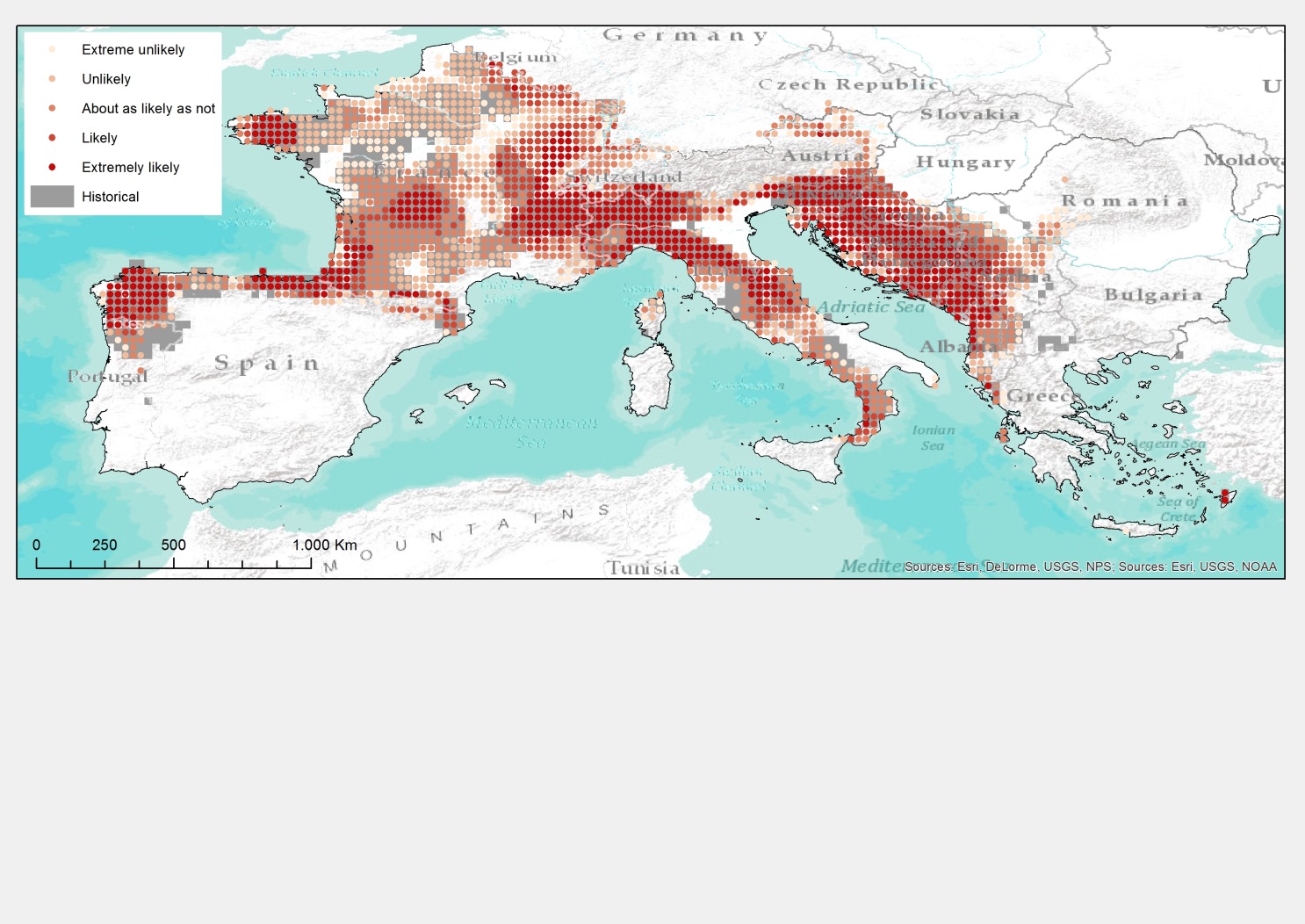
**

**Figure S6** Future likelihood suitability map for Castanea (2070)

**
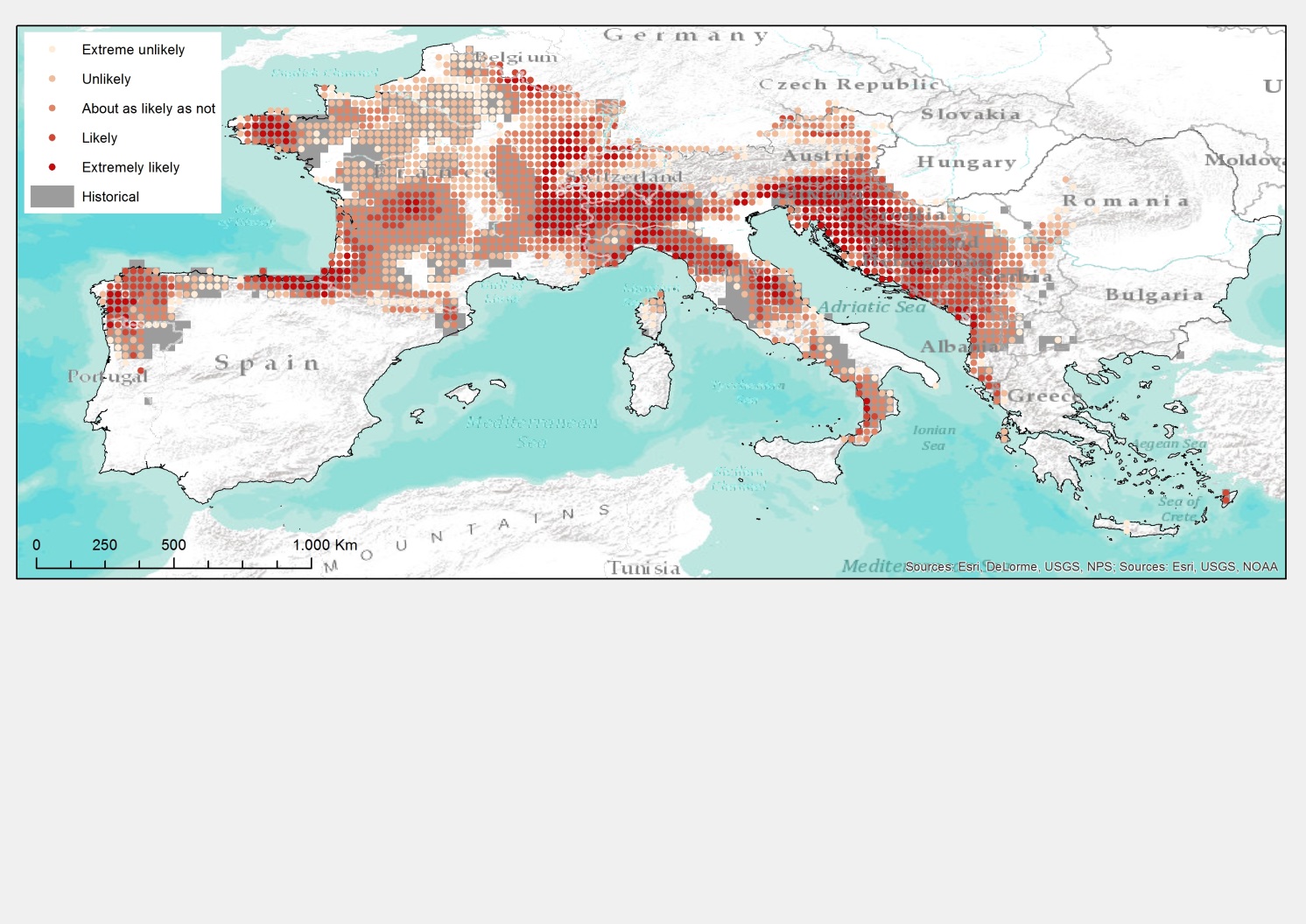
**

**Figure S7** Future likelihood suitability map for Larix (2050)

**
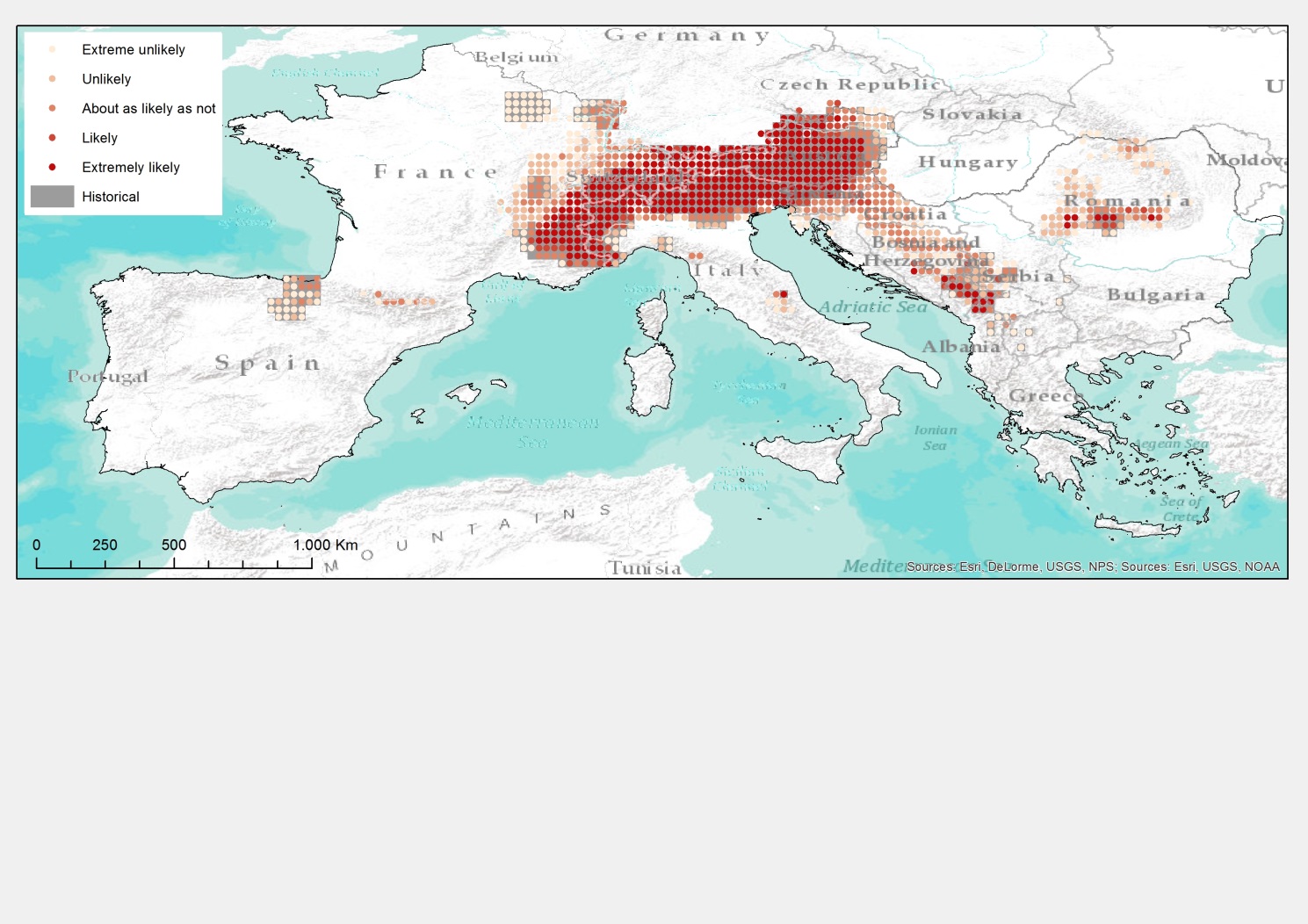
**

**Figure S8** Future likelihood suitability map for Larix (2070)

**
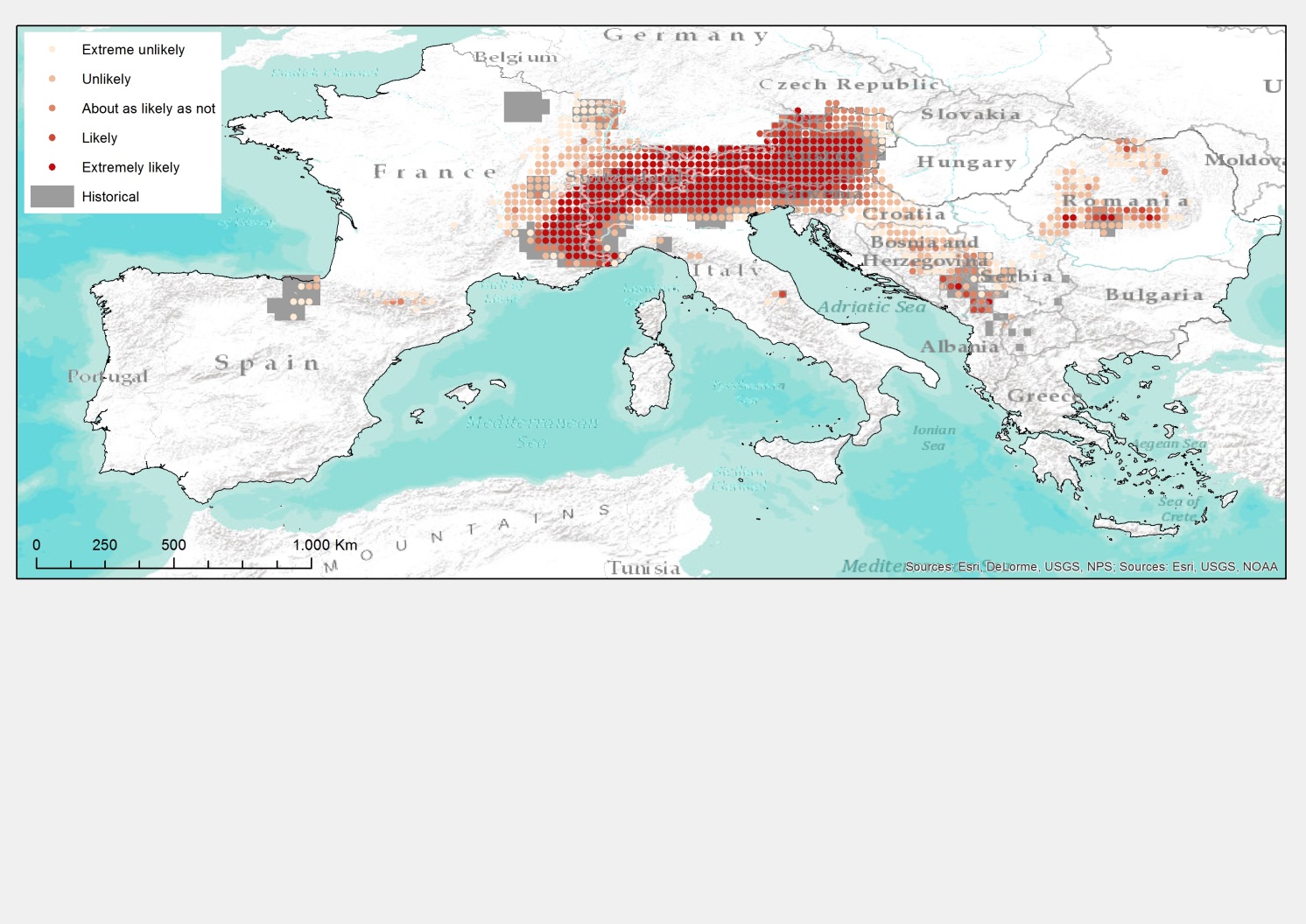
**

**Figure S9** Future likelihood suitability map for Picea (2050)

**
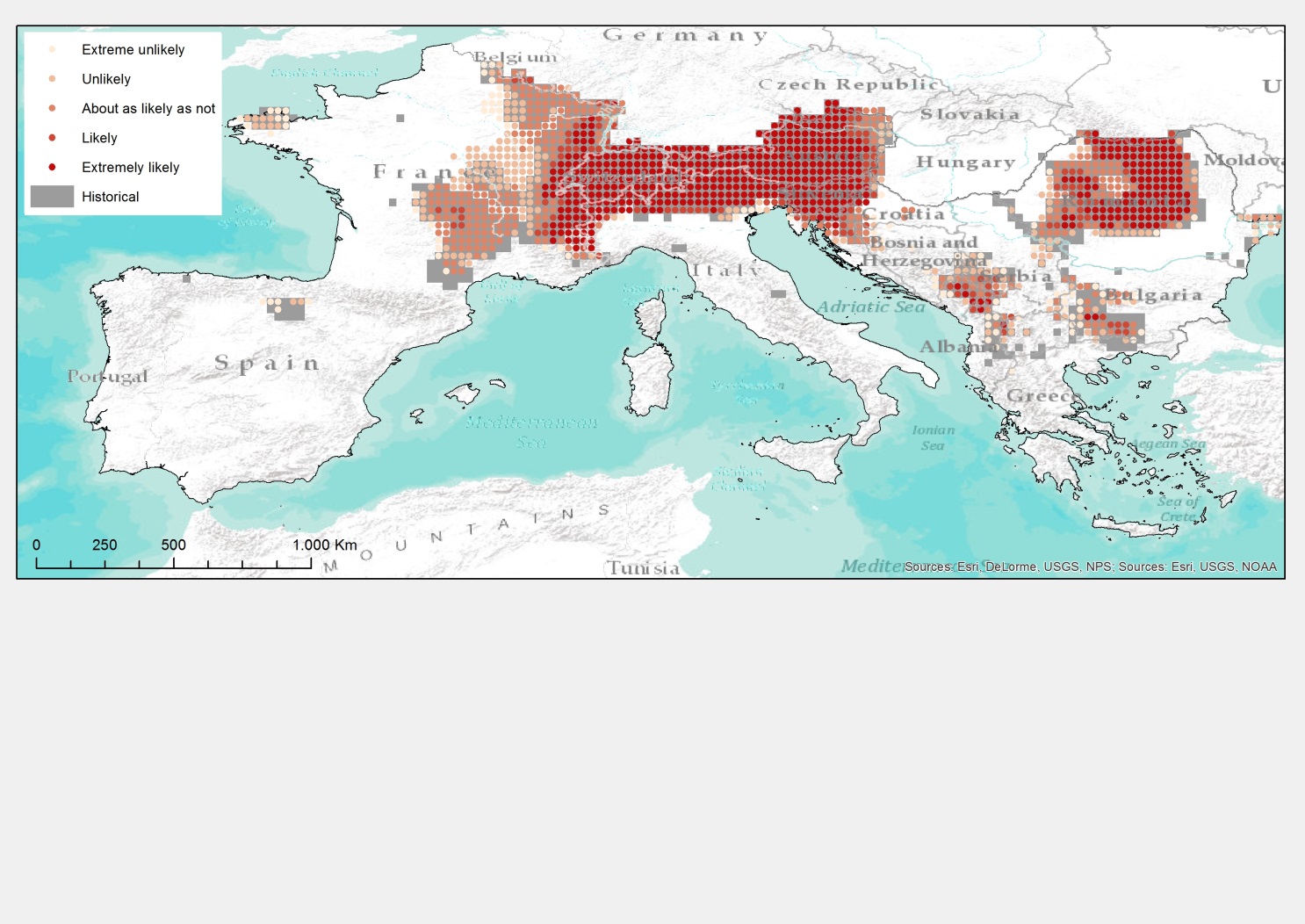
**

**Figure S10** Future likelihood suitability map for Picea (2070)

**
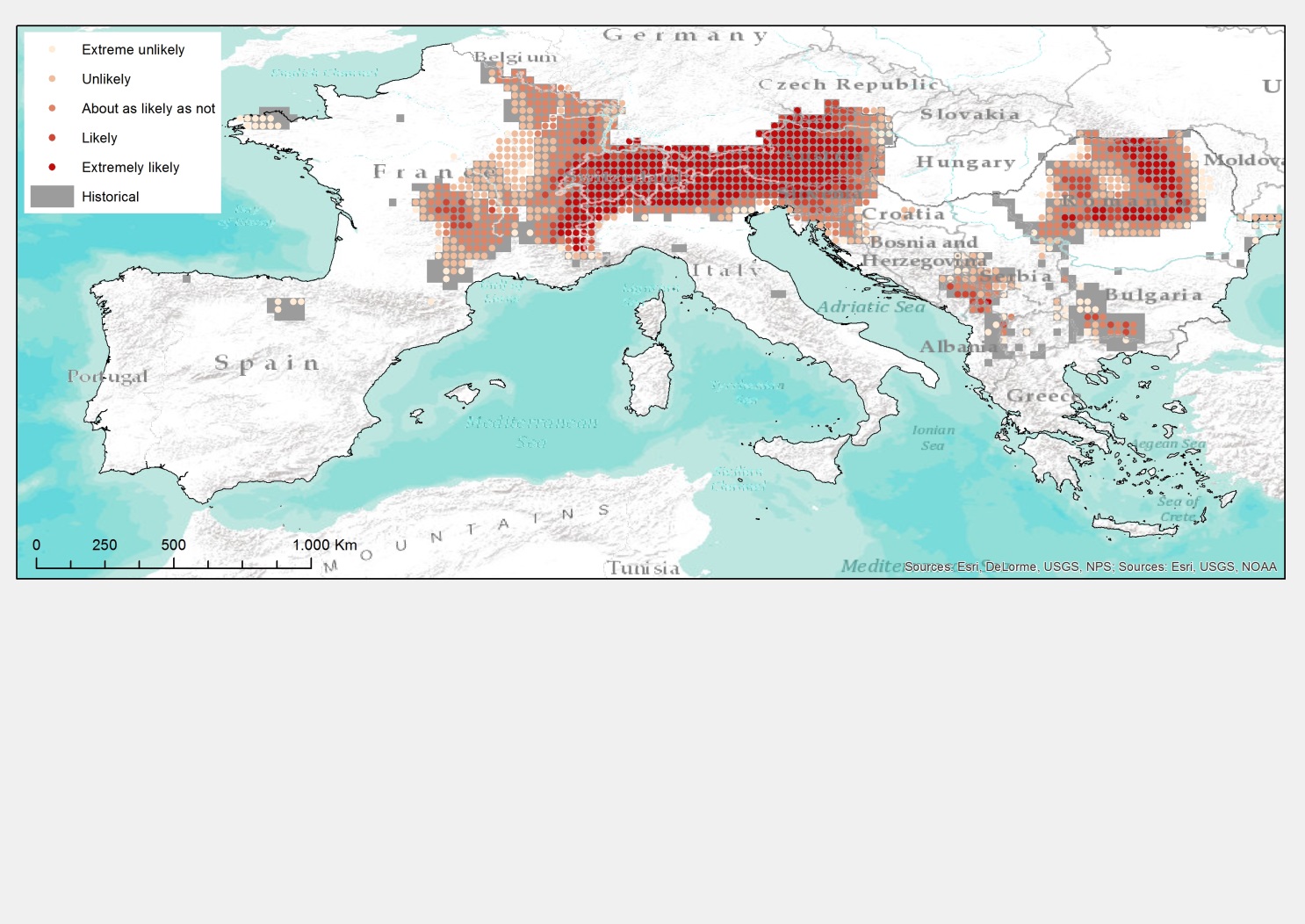
**

**Figure S11** Future likelihood suitability map for PinusPin (2050)


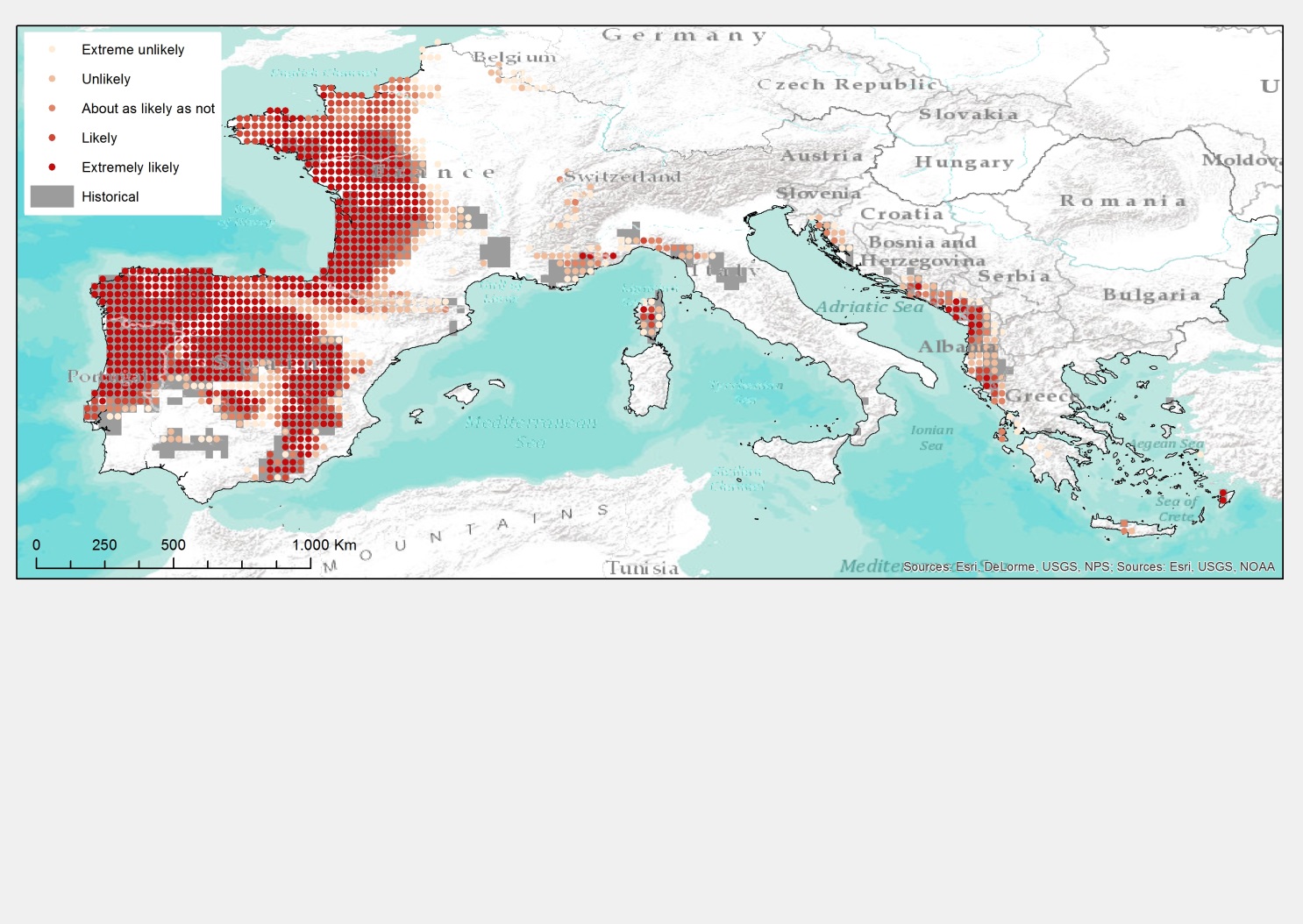


**Figure S12** Future likelihood suitability map for PinusPin (2070)

**
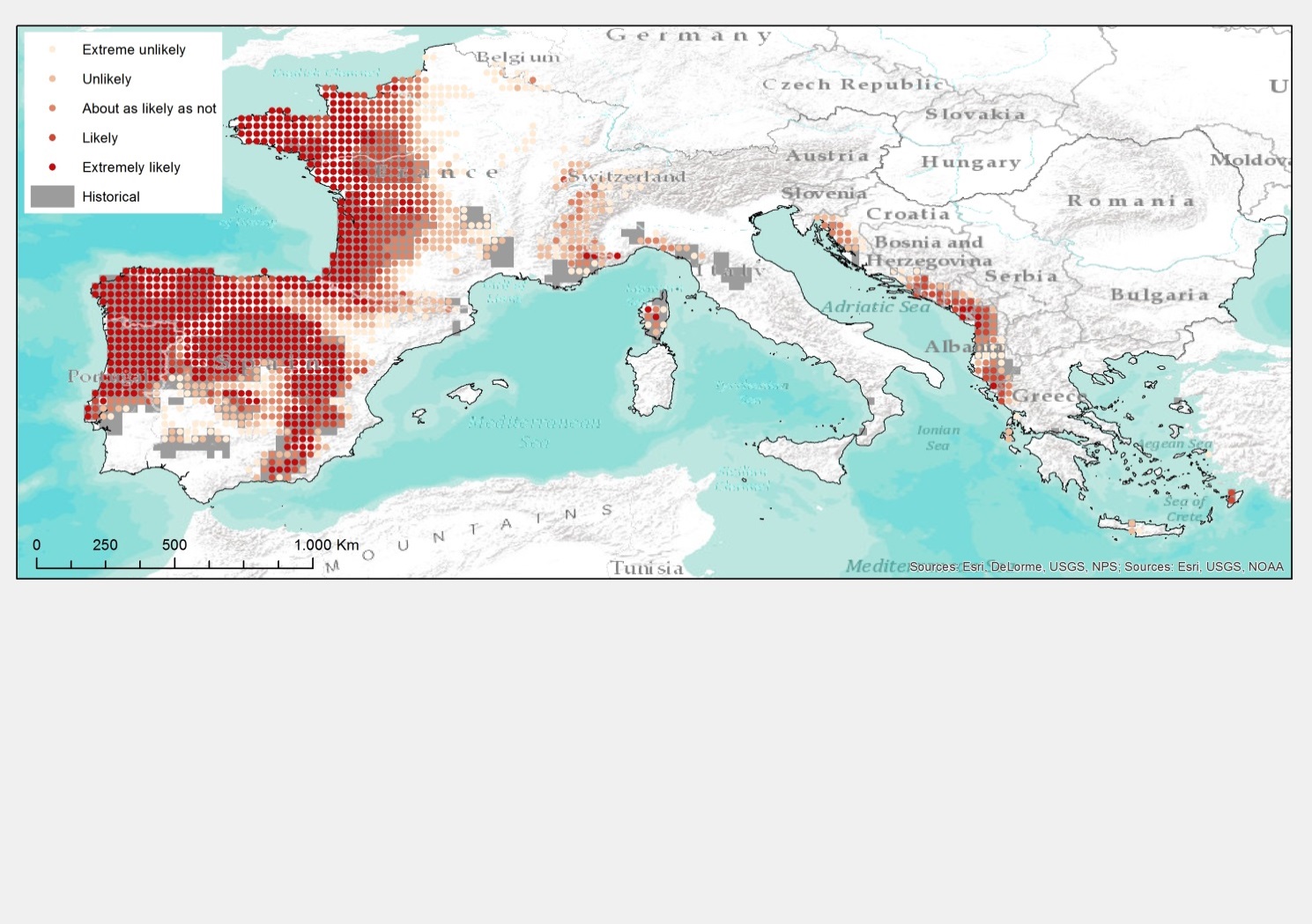
**

**Figure S13** Future likelihood suitability map for PinusSylv (2050)

**
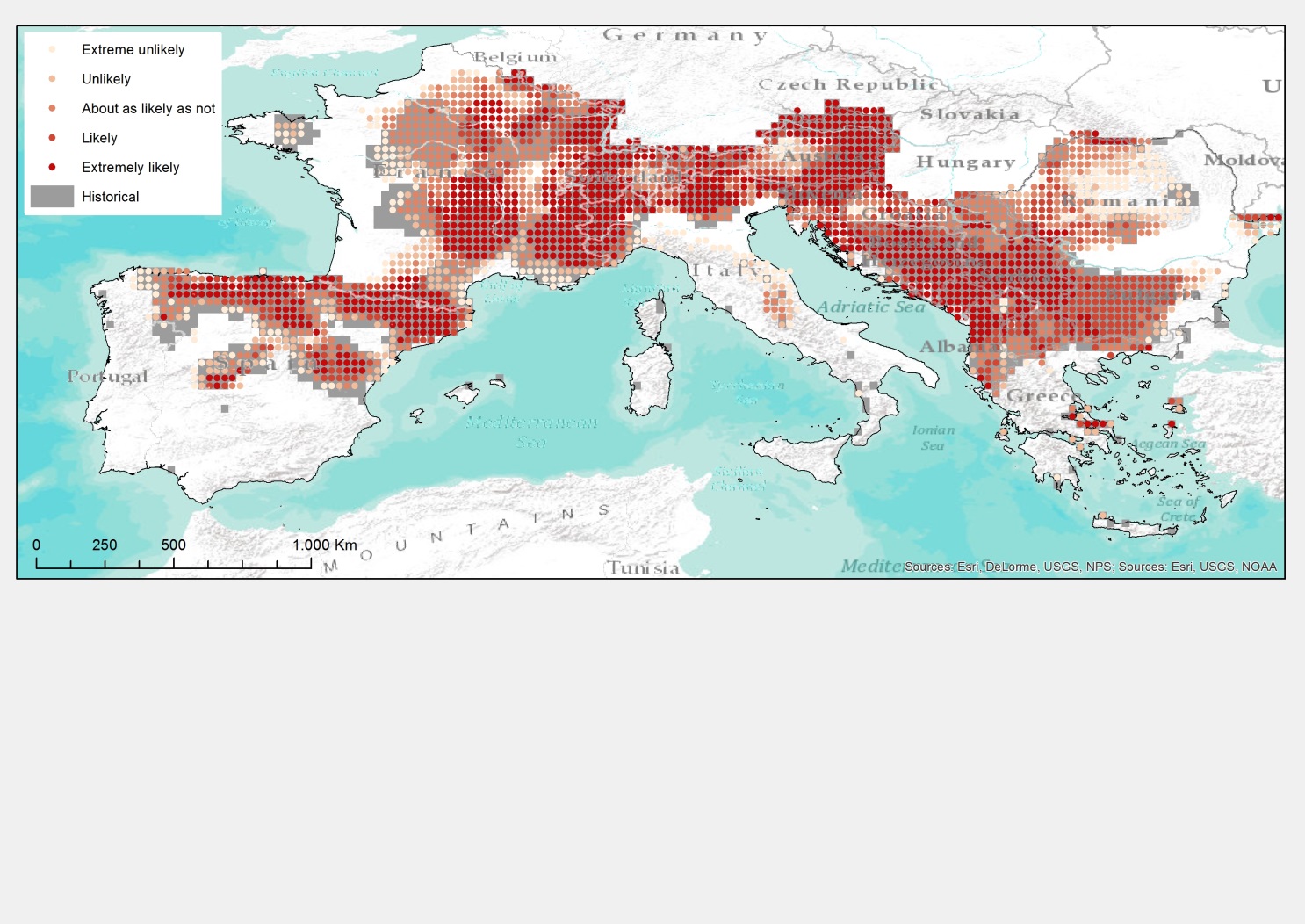
**

**Figure S14** Future likelihood suitability map for PinusSylv (2070)

**
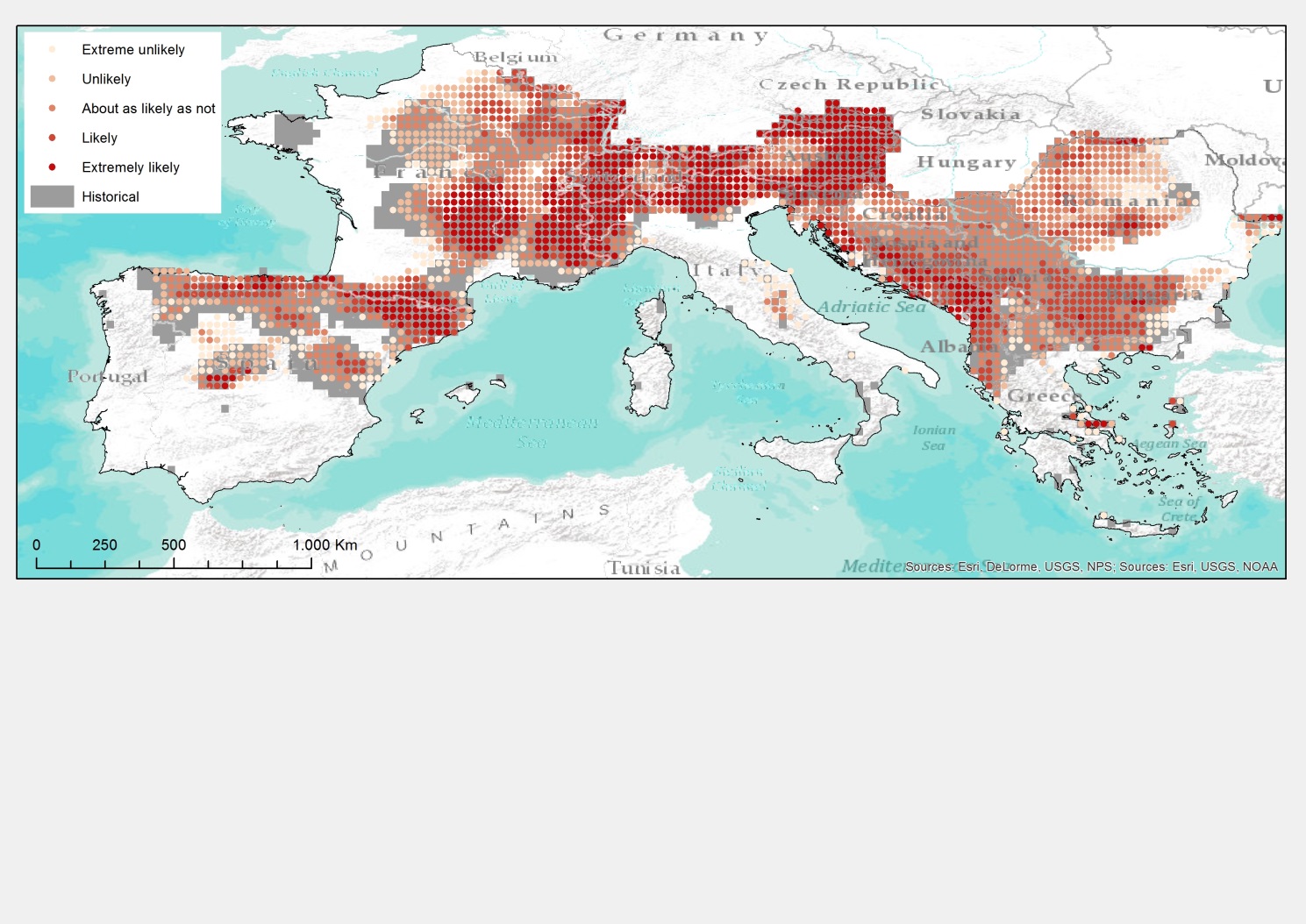
**

**Figure S15** Future likelihood suitability map for QuercusRP (2050)

**
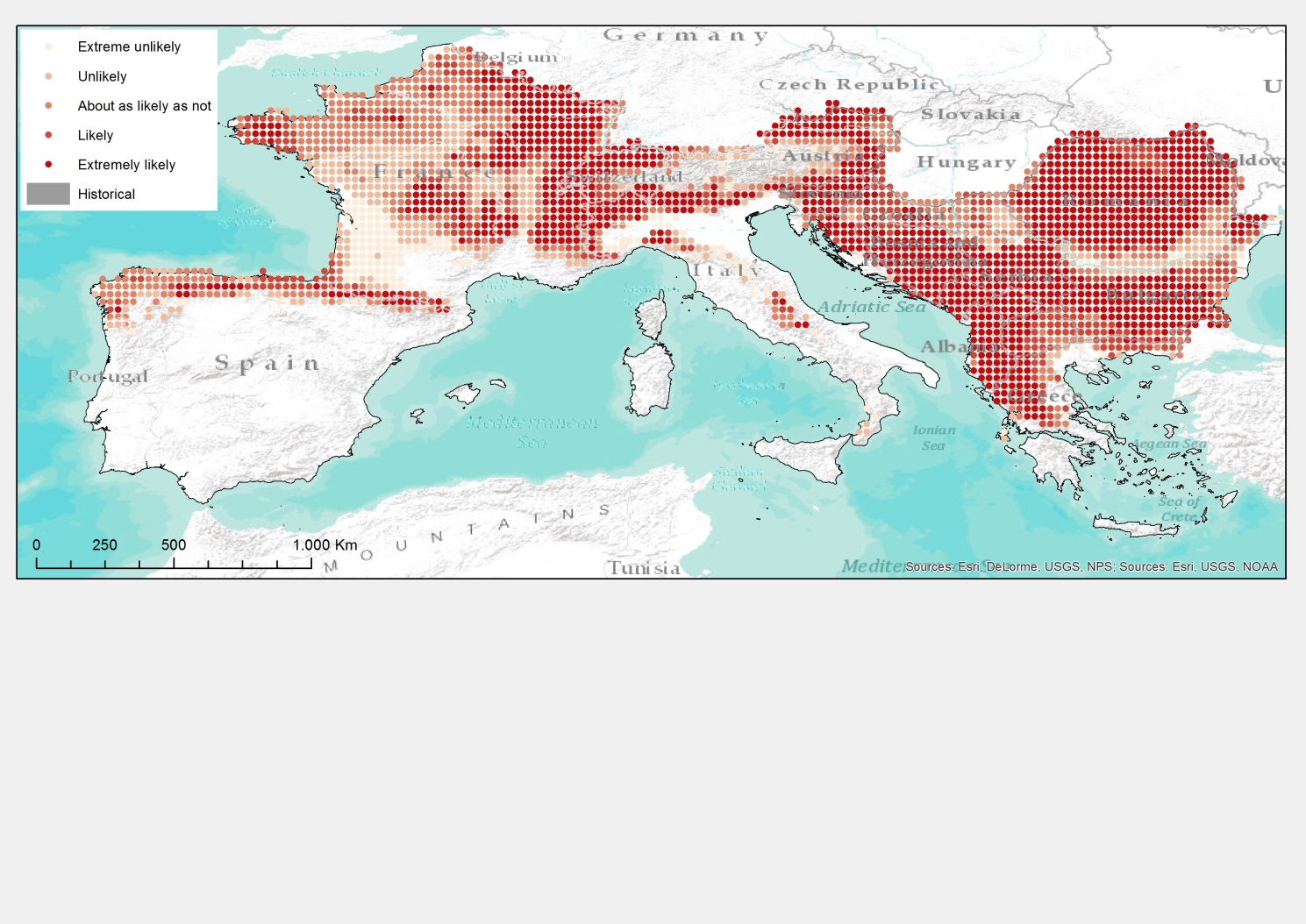
**

**Figure S16** Future likelihood suitability map for QuercusRP (2070)

**
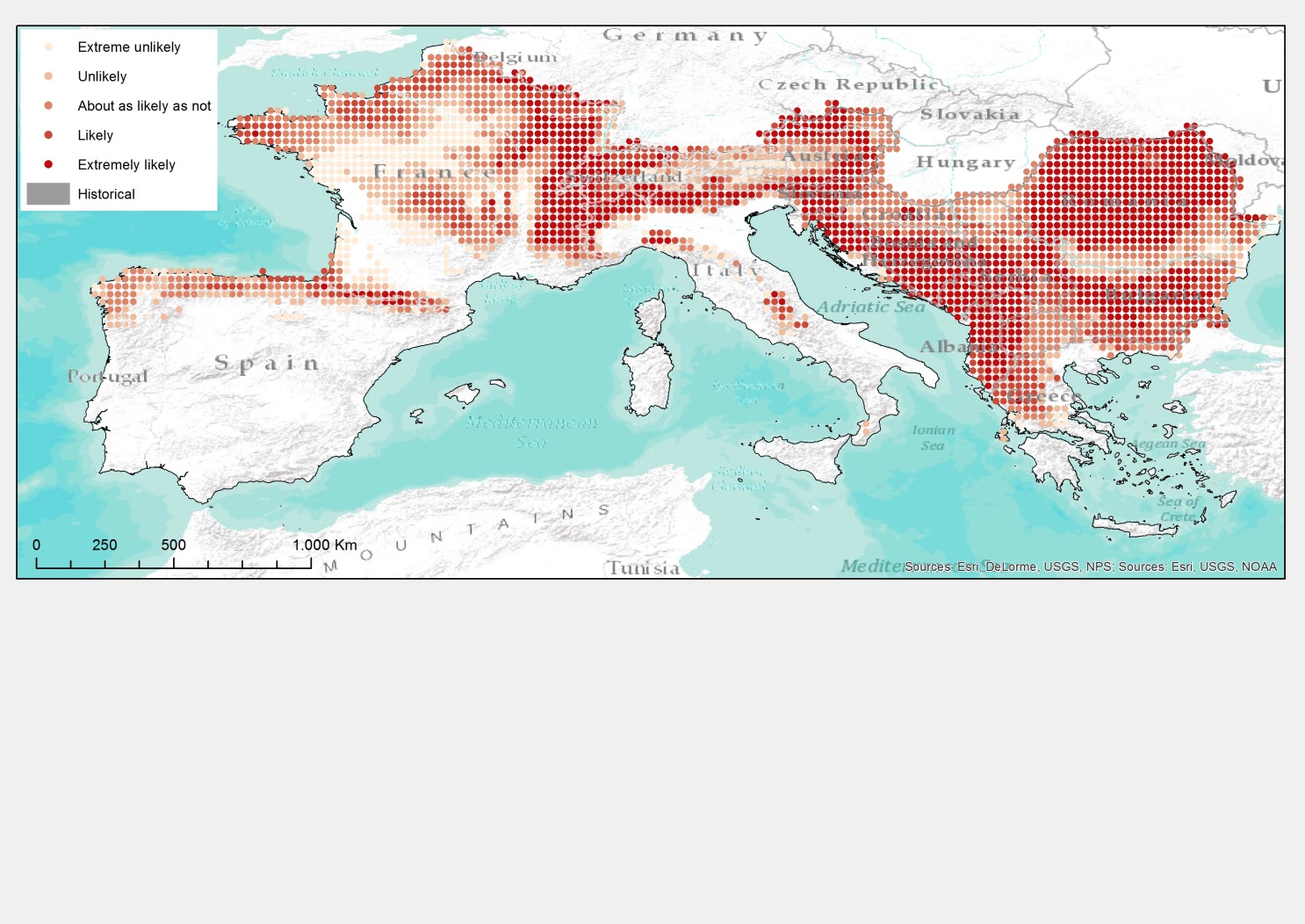
**
