# Supplementary material for: Likelihood of changes in forest species suitability, distribution, and diversity under future climate: The case of Southern Europe
Source: Ecol Evol. 2017 Oct 7;7(22):9358–75. doi: 10.1002/ece3.3427 (PMC5696419; doi:10.1002/ece3.3427)

**Supporting Information 2**

**Appendix S2** Plots representing changes in forest distribution in number of pixels divided into sub-regions. Different capital letters mean that distributions are significantly different at the 99.9% level (p-value < 0.001)

**Figure S17** Alpine Sub-Region. Changes in forest distribution in number of pixels. Different capital letters mean that distributions are significantly different at the 99.9% level (p-value < 0.001).


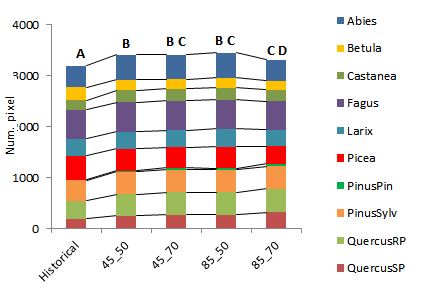


**Figure S18** Atlantic Sub-Region. Changes in forest distribution in number of pixels. Different capital letters mean that distributions are significantly different at the 99.9% level (p-value < 0.001).


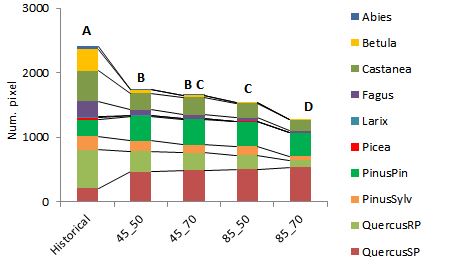


**Figure S19** Continental Sub-Region. Changes in forest distribution in number of pixels. Different capital letters mean that distributions are significantly different at the 99.9% level (p-value < 0.001).


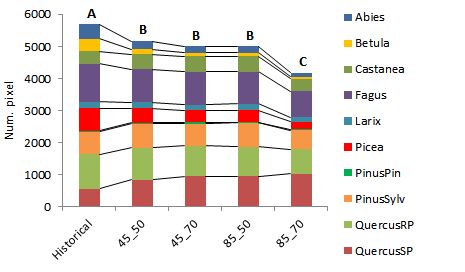


**Figure S20** Mediterranean Sub-Region. Changes in forest distribution in number of pixels. Different capital letters mean that distributions are significantly different at the 99.9% level (p-value < 0.001).


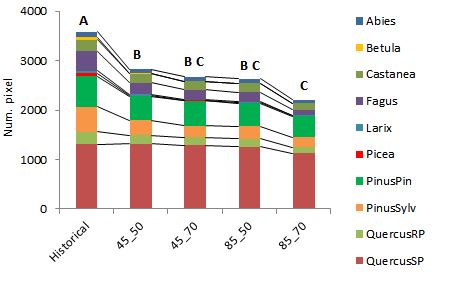

Supplement: Supplementary file 2 [file ECE3-7-9358-s002.docx]
