# Supplementary material for: Likelihood of changes in forest species suitability, distribution, and diversity under future climate: The case of Southern Europe
Source: Ecol Evol. 2017 Oct 7;7(22):9358–75. doi: 10.1002/ece3.3427 (PMC5696419; doi:10.1002/ece3.3427)

**Supporting Information 3**

**Appendix S3** Plots representing changes in forest diversity (expressed in number of categories for each pixel) divided into sub-regions. Different capital letters mean that distributions are significantly different at the 99.9% level (p-value < 0.001)

**Figure S21** Atlantic Sub Region. Changes in forest diversity. Bordered portions of bars represent the most frequent combination of categories. Different capital letters mean that distributions are significantly different at the 99.9% level (p-value < 0.001)


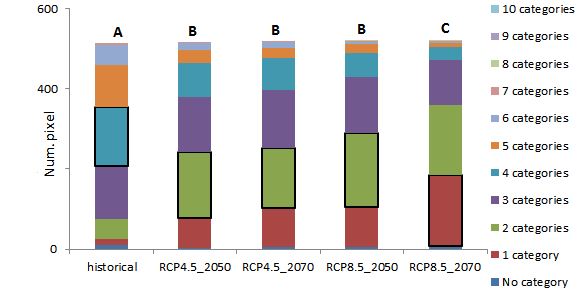


**Figure S22** Continental Sub Region. Changes in forest diversity. Bordered portions of bars represent the most frequent combination of categories. Different capital letters mean that distributions are significantly different at the 99.9% level (p-value < 0.001)


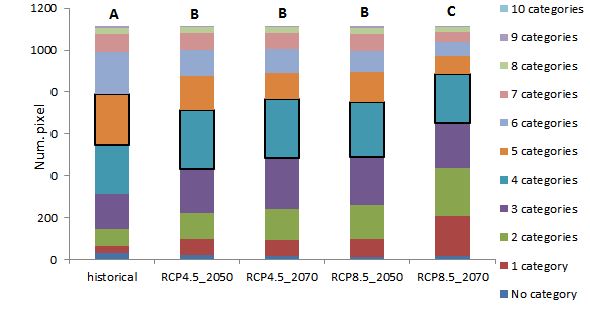


**Figure S23** Mediterranean Sub Region. Changes in forest diversity. Bordered portions of bars represent the most frequent combination of categories. Different capital letters mean that distributions are significantly different at the 99.9% level (p-value < 0.001)


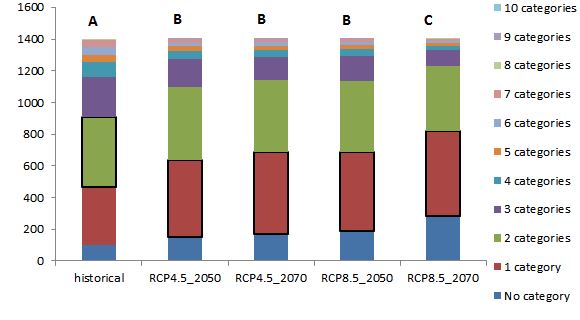


**Figure S24** Alpine Sub Region. Changes in forest diversity. Bordered portions of bars represent the most frequent combination of categories. Different capital letters mean that distributions are significantly different at the 99.9% level (p-value < 0.001)


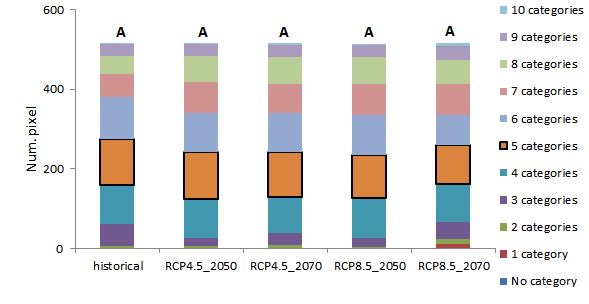

Supplement: Supplementary file 3 [file ECE3-7-9358-s003.docx]
